# Transcriptome landscape of *Rafflesia cantleyi* floral buds reveals insights into the roles of transcription factors and phytohormones in flower development

**DOI:** 10.1371/journal.pone.0226338

**Published:** 2019-12-18

**Authors:** Safoora Amini, Khadijah Rosli, Mohd-Faizal Abu-Bakar, Halimah Alias, Mohd-Noor Mat-Isa, Mohd-Afiq-Aizat Juhari, Jumaat Haji-Adam, Hoe-Han Goh, Kiew-Lian Wan

**Affiliations:** 1 School of Biosciences and Biotechnology, Faculty of Science and Technology, Universiti Kebangsaan Malaysia, UKM Bangi, Selangor, Malaysia; 2 Centre for Biotechnology and Functional Food, Faculty of Science and Technology, Universiti Kebangsaan Malaysia, UKM Bangi, Selangor, Malaysia; 3 Malaysia Genome Institute, Jalan Bangi, Kajang, Selangor, Malaysia; 4 School of Environmental and Natural Resource Sciences, Faculty of Science and Technology, Universiti Kebangsaan Malaysia, UKM Bangi, Selangor, Malaysia; 5 Institute of Systems Biology, Universiti Kebangsaan Malaysia, UKM Bangi, Selangor, Malaysia; University of Naples Federico II, ITALY

## Abstract

*Rafflesia* possesses unique biological features and known primarily for producing the world’s largest and existing as a single flower. However, to date, little is known about key regulators participating in *Rafflesia* flower development. In order to further understand the molecular mechanism that regulates *Rafflesia cantleyi* flower development, RNA-seq data from three developmental stages of floral bud, representing the floral organ primordia initiation, floral organ differentiation, and floral bud outgrowth, were analysed. A total of 89,890 transcripts were assembled of which up to 35% could be annotated based on homology search. Advanced transcriptome analysis using K-mean clustering on the differentially expressed genes (DEGs) was able to identify 12 expression clusters that reflect major trends and key transitional states, which correlate to specific developmental stages. Through this, comparative gene expression analysis of different floral bud stages identified various transcription factors related to flower development. The members of WRKY, NAC, bHLH, and MYB families are the most represented among the DEGs, suggesting their important function in flower development. Furthermore, pathway enrichment analysis also revealed DEGs that are involved in various phytohormone signal transduction events such as auxin and auxin transport, cytokinin and gibberellin biosynthesis. Results of this study imply that transcription factors and phytohormone signalling pathways play major role in *Rafflesia* floral bud development. This study provides an invaluable resource for molecular studies of the flower development process in *Rafflesia* and other plant species.

## Introduction

*Rafflesia* is a member of the holoparasitic plant family, Rafflesiaceae, which is known to produce the world’s largest flower. There are over 30 species of *Rafflesia* that can be found in the tropical rainforest of Southeast Asia. *Rafflesia cantleyi* was the first species identified from Peninsular Malaysia, with more species identified later on [1–2]. Besides an extraordinary flower size, the floral structure of *R*. *cantleyi* is highly modified compared to other angiosperms. It has no apparent leaves, stems or roots, and only appears as a flower, which parasitises a specific host, *Tetrastigma* [3]. *Rafflesia* possesses five perigone lobes as perianth connected to a diaphragm enclosing a large and bowl-shaped floral chamber with a central column as the reproductive organ [4]. Apart from gigantism, flowering of *R*. *cantleyi* is irregular, infrequent, and the development of floral bud takes up to nine months. At the early developmental stage, the swollen bud of *R*. *cantleyi* appears through the bark of *Tetrastigma* covered with bracts and continues to grow progressively. Upon maturation and bracts abscission, the bud opens gradually over a 24 to 48-hour period [5].

Flower development, preceded by the flowering process is the most important developmental event in a plant’s life cycle. Molecular and genetic studies in the annual model species *Arabidopsis* present an intricate genetic network that orchestrates the flowering process, controlled by diverse exogenous and endogenous factors. Endogenous factors include hormones, autonomous pathway, and aging pathway, whereas exogenous factors comprise of photoperiod and vernalisation [6]. Subsequently, genes involved in the flowering pathway converge on floral integrators to activate floral meristem identity genes, which are essential for floral organ development. Floral integrators include *FLOWERING LOCUS T* (*FT*), *CONSTANS* (*CO*), *CONSTANS-LIKE* (*COL*), *SUPPRESSOR OF OVEREXPRESSION OF CO1* (*SOC1*), *FLOWERING LOCUS C* (*FLC*), as well as floral meristem identity genes, including *LEAFY* (*LFY*) and *APETALA1* (*AP1*) [7]. Floral integrators activate floral organ identity genes known as ABC (extended to ABCDE) model genes [8]. Additionally, transcription factors (TFs) such as bHLH, MYB, and MADS-box families are important regulatory proteins of transcription in flower development [9].

Substantial progress has been achieved in understanding the mechanism of flower development, particularly in Arabidopsis [8, 10]. However, while considerable work has been carried out in recent years on the different aspects of *Rafflesia* [11–12] including genes potentially involved in the growth and flower development [13–14], our knowledge regarding the molecular mechanism of *R*. *cantleyi* floral development is still very limited due to scarce sample availability, challenges during sample collection, lack of suitable material in the wild, undeveloped analytical methodologies, and inadequate molecular resources. In an effort to address this issue, we have previously generated RNA-seq data from early, mid and advanced *R*. *cantleyi* bud stages [15]. The cross-sections of these three bud stages showed the early (floral bud stage 1), mid (floral bud stage 2) and advanced (floral bud stage 3) developmental stages. Floral bud stage 1 (FBS1) consists of undifferentiated cells while floral bud stage 2 (FBS2) contains moderately differentiated and visible internal organs, whereas floral bud stage 3 (FBS3) have more developed and mature internal organs. In this study, functional annotation of the transcriptome data and differential gene expression analysis were carried out to glean insights into molecular genetics underlying the regulation of flower development in *R*. *cantleyi*. This valuable genomic resource will facilitate further study of flower development in *Rafflesia* and other plant species.

## Materials and methods

### Transcriptome *de novo* assembly and functional annotation

Raw data previously generated as described in [15] were used in this study. The raw data obtained for three different floral bud stages (early, mid and advanced) has been registered under NCBI BioProject with the accession number PRJNA378435. The raw reads were trimmed and quality-filtered using Trimmomatic [16]. The high-quality raw reads were pooled for *de novo* assembly using the Trinity (v2.0.6) analysis pipeline [17]. The generated contigs were assembled and constructed sequences that could not be extended on either end are considered as unique transcripts [18], herein referred as transcripts. All the assembled transcripts were subjected to BLASTX similarity search against the NCBI non-redundant (Nr) (www.ncbi.nlm.nih.gov) and Swiss-Prot (www.uniprot.org) databases with an E-value cut-off of 1e^-5^. Based on Nr BLAST result, BLAST2GO [19] was performed to obtain Gene Ontology (GO) annotation of assembled transcripts to represent biological process, cellular component, and molecular function categories. WEGO [20] was employed to retrieve the GO functional classification to explain the distribution of gene functions. Transcript sequences were also searched against the Cluster of Orthologous Groups (COG) protein database (www.ncbi.nlm.nih.gov/COG/). Furthermore, annotated transcripts were searched against the Kyoto Encyclopedia of Genes and Genomes (KEGG) pathway maps database [21] for mapping of biological pathways represented by the *R*. *cantleyi* bud transcriptome. Protein coding sequences of *R*. *cantleyi* transcriptome were predicted via Transdecoder (transdecoder.github.io) implemented in the Trinity analysis pipeline. Trinotate annotation pipeline (trinotate.github.io) was carried out to annotate the transcriptome against Pfam [22–23].

### Identification of transcription factors

The transcripts were aligned against known TFs, as grouped in Plant Transcription Factor Database (PlnTFDB) [24], using default parameters and cut-off E-value of 1e^-5^. PlnTFDB is a comprehensive library of plant TFs that provides the complete lists of TFs families of fully sequenced genomes. Online protein sequence data for all genes listed in PlntTFDB version 3.0 (plntfdb.bio.uni-potsdam.de/v3.0/) were downloaded for transcript annotation using BLASTX. Heatmaps depicting the expression patterns of TF families at different bud stages of *R*. *cantleyi* were created using the MeV tool (www.tm4.org/mev.html).

### Differential gene expression analysis

To compare the differences in gene expression between different bud stages, transcript abundance was estimated from mapped reads with RSEM [25]. The relative gene expression levels for each bud samples were normalised and expressed as Fragments per Kilobase of Exon per Million Reads Mapped (FPKM) values. Bioconductor package edgeR [26] was used to determine the DEGs by evaluating the dispersion of the entire dataset. Significant DEGs were defined by P-value ≤ 0.001, Benjamin-Hochberg False Discovery Rate (FDR) ≤ 0.05 and |Log_2_ fold change| > 2.

### KEGG pathway and GO enrichment analysis of DEGs

The pathway enrichment analysis with hypergeometric test was performed using ‘Annotate’ and ‘Identify’ programs in KEGG Orthology-Based Annotation System (KOBAS 2.0) with Benjamini-Hochberg FDR correction [27] to annotate putative pathways and biological functions of DEGs. The web-based ReviGO software (revigo.irb.hr) was used to reveal enriched gene GO functional categories identified from DEGs [28].

### Data validation by RT‑qPCR

*R*. *cantleyi* buds of different developmental stages (early mid, and advanced) for RT-qPCR were sampled independently from the Forest Reserve in Raub, Pahang, Malaysia (3° 47′ 24″ N, 101° 51′25″E). Samples were rinsed using 10% (v/v) Clorox® solution (1% sodium hypochlorite) after carefully dissected from the host plant, followed by three rinses with sterile water, and flash frozen in liquid nitrogen before stored at -80°C. Inner tissues (for FBS2 and FBS3 perigone lobe) of the floral buds were used for the isolation of total RNA using modified CTAB extraction protocol [29–31]. RNA quality and quantity were evaluated using gel electrophoresis, ND-1000 Nanodrop spectrophotometer (Thermo Scientific) and Agilent 2100 Bioanalyzer with a minimum RNA integrity number of 7. Using PrimerBlast 5.0, primers were designed to amplify short regions for each target and reference genes ranging in product size from 90 to 120 bp. The primer sequences of references genes and 12 selected genes are listed in S1 Table. For each sample, one-step RT-qPCR was performed on the BioRad CFX96 TouchTM Deep Well Real-Time PCR, using the QuantiNov a® SYBR® Green RT-PCR kit (Qiagen, USA) with 1 μg DNase-treated (RNA-free DNase, Qiagen, USA) RNA. The amplification was carried out with the following cycling programme: 30 min at 50°C, 30 s at 95°C, 39 cycles of 30 s at 60°C, and 30 s at 76°C. Data were analysed with BioRad CFX Manager software (version 1.3). To determine the relative fold change differences for each sample, the C_T_ value of each candidate genes was normalised to the C_T_ values of two reference genes *GLYCERALDEHYDE-3-PHOSPHATE DEHYDROGENASE* (*GAPDH*) (TR42340|c0_g6_i1) and *UBIQUITIN5* (*UBQ5*) (TR35146|c0_g3_i1) based on the comparative C_T_ (2^-ΔΔCT^) method (Schmittgen and Livak, 2008). Relative expression in RNA-seq was confirmed by three independent biological replicates along with respective three technical replicates.

## Results

### Morphological characteristics of *R*. *cantleyi* floral buds

**Fig 1:**
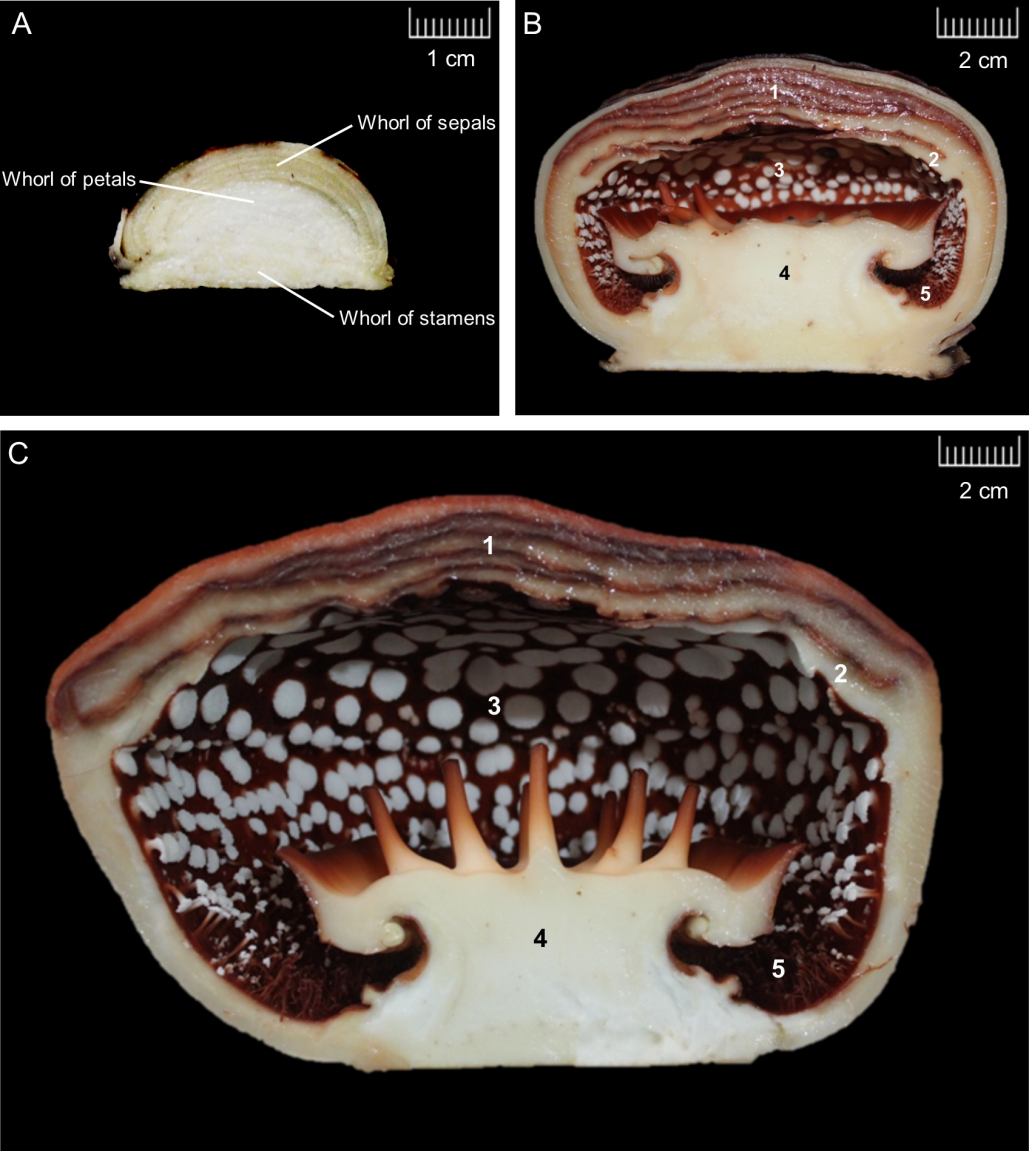
Morphology of *Rafflesia cantleyi* floral bud developmental stages. Longitudinal section of (A) floral bud stage 1 with the initiation of reproductive development, (B) floral bud stage 2 of with organ differentiation and outgrowth, and (C) floral bud stage 3 with fully developed floral organs for blooming. Structures identified are perianth lobes (1), diaphragm (2), floral chamber (3), reproductive column (4) and ramenta (5).

The morphological changes in *R*. *cantleyi* floral buds were examined and three bud stages were proposed, which correspond to floral organ primordia initiation, floral organ differentiation, and floral bud outgrowth (Fig 1). At FBS1, bud appears on the stem while the *Tetrasigma* bark still cover the bud. In the undifferentiated *R*. *cantleyi* bud, three whorls can be distinguished. First whorl of five perianths (perigone) lobes is derived from the outer whorl organ primordia (sepals), the diaphragm is derived from the middle whorl (petals), whereas the reproductive column is derived from the inner or third whorls (stamens). The longitudinal section of FBS1 indicates control of cell proliferation, expansion, and growth in the floral meristem during the early stage of floral organ primordia initiation (Fig 1A). Sepal, petal, and stamen primordia cells are arranged in concentric rings, in a similar robust pattern that can be observed during floral organ determination in eudicot flowers. In this study, FBS1 is defined at the time point when petal primordia and stamen primordia are visible while sepal primordia covering the rest of the meristem. Based on our observations, flower primordia were initiated before FBS1 as the rapid coordinated burst of cell division and expansion already occurred, generating a group of cells as a spherical flower primordium, from which all floral tissues are derived.

FBS2 represents the initiation of floral organ separation and specification (Fig 1B). The bud was covered with bracts, which turned dark brown towards the end of this stage. Bracts are present throughout floral bud development and have a protective function [32]. The differentiation of reproductive column and perianth lobes is notable, while ramenta, a series of multicellular, vascularised and branched structures, are visible at the inner surface of the perianth tube. The five fused-perianth lobes (connation) elongate and overlap, covering the developing stamens. The perianth lobes emerge gradually in a spiral and together with the diaphragm grow above the floral apex, which form the perianth tube. Following the appearance of the diaphragm, stamens are initiated on the flanks of the floral apex [14]. With progressive growth, the change in floral color is also obvious. The diaphragm in *R*. *cantleyi* bud is the lowest layer beneath the perianth lobes.

FBS3 is the later stage of floral organ growth. The pre-anthesis stage in *R*. *cantleyi* is when bracts detach and the outer side of perianth lobes becomes visible (Fig 1C). In advanced floral buds, all organs lengthened with more pronounced elongation of perianth lobes, while fused diaphragm-perianth lobes (adnation) still appear morphologically similar and can only be identified based on their position in perianth tube. At this stage, the floral chamber is fully developed. Anthesis in *R*. *cantleyi* only occurs when all floral organs are fully grown and differentiated.

### Functional annotation and classification of *R*. *cantleyi* bud transcriptome

A total of 91,638,836 clean reads were obtained from the three different developmental stages and assembled into 89,690 transcripts with an N50 of 1,653 bp and 20,592 predicted protein sequences. Results of functional prediction and classification are summarised in S2 Table. Based on sequence similarity search against the NCBI Nr protein database and the Swiss-Prot protein sequence database, 31,444 transcripts (35%) and 21,781 transcripts (24.3%) were annotated, respectively. Furthermore, 5,850 transcripts (6.5%) were annotated using the KEGG database and 5,069 transcripts (5.6%) by the Pfam database. The highest number of *R*. *cantleyi* transcripts showed similarity to sequences from *Vitis vinifera* (23%), followed by *Ricinus communis* (19.5%), *Populus trichocarpa* (19%), and *Theobroma cacao* (9%) (S1A Fig). According to the E-value distribution of significant hits against the Nr database, 61.7% of the matched sequences showed E-value ≤ 1.0 E-50 (S1B Fig). Other than that, the similarity distribution of the top Nr BLAST hits revealed that similarity scores of more than 57% of matched sequences were higher than 70%, while 27% of transcripts showed similarity ranging from 50% to 70% (S1C Fig).

**Fig 2:**
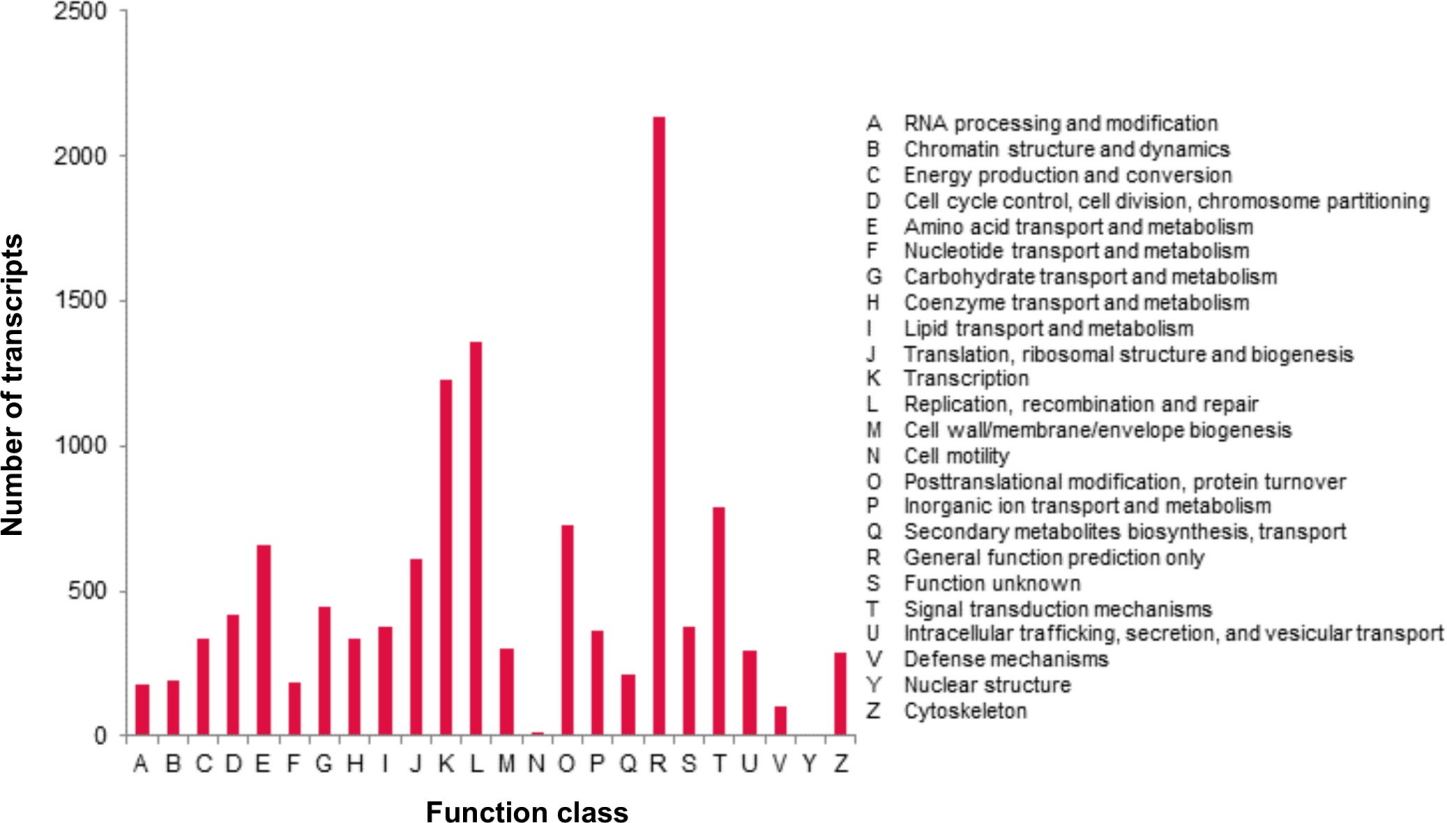
COG classification of *Rafflesia cantleyi* floral bud transcripts. A total of 7,172 transcripts were assigned to 25 COG categories. The y-axis indicates the number of transcripts in each category.

A total of 7,172 (7.9%) transcripts were assigned to 25 COG categories. Of these, the largest category was ‘general function prediction only’ (2,134), followed by ‘replication, recombination and repair’ (1,361), ‘transcription’ (1,226), ‘signal transduction mechanisms’ (788); whereas the categories of ‘nuclear structure’ (3) and ‘cell motility’ (13) represented the smallest classifications (Fig 2). These assigned functions of transcripts covered a wide range of COG classifications, indicating that the floral bud transcriptome represented a broad variety of transcripts in *R*. *cantleyi*. These include two COG terms related to flower development, ‘cell cycle control, cell division’ and ‘signal transduction mechanism’ (S2 Fig).

**Fig 3:**
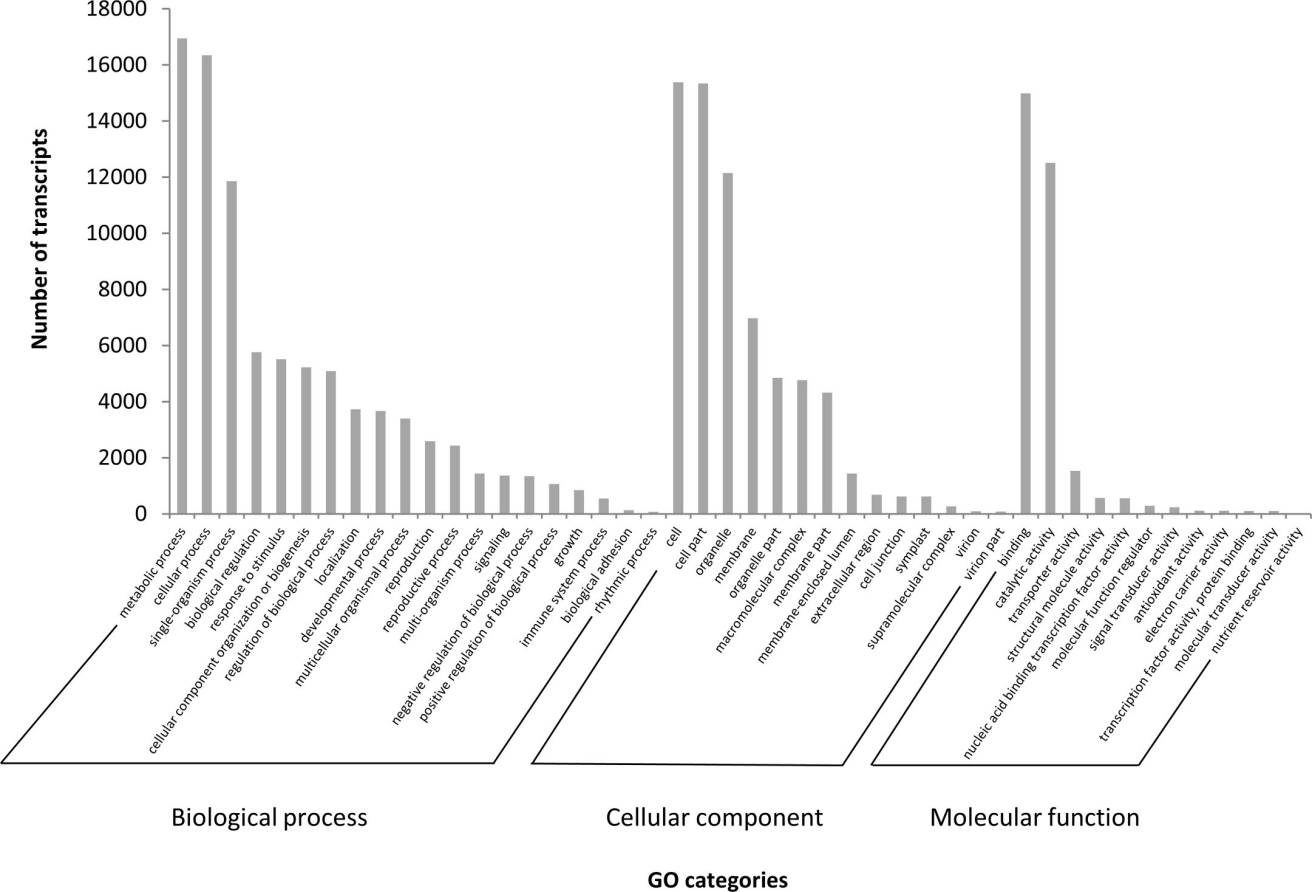
Gene Ontology functional classification of *Rafflesia cantleyi* floral bud transcriptome. The results are summarised in three GO categories: biological process, molecular function and cellular component. The y-axis indicates the number of transcripts in each category.

For GO term assignment, 28,992 (32.3%) transcripts were classified into 46 GO categories of 20 biological processes, 14 cellular components, and 12 molecular functions (Fig 3, S3 Table). Under the biological process category, ‘metabolic process’ (16,950), ‘cellular process’ (16,346), and ‘single-organism process’ (11,855) were prominently represented. Within cellular components, ‘cell’ (15,385), ‘cell part’ (15,345), and ‘organelle’ (12,145) were the most highly represented categories. For the molecular function category, the largest proportion of transcripts was grouped into ‘binding’ (14,992) and ‘catalytic activity’ (12,510). Additionally, only a few sequences were assigned to ‘rhythmic process’, ‘virion’, ‘virion part’ and ‘nutrient reservoir activity’ terms with less than one hundred sequences each.

Pathway-based analysis provides information on molecular regulation in response to environmental and developmental changes. Generally, transcripts in the same pathway confer similar biological functions. In total, there were 5,850 transcripts mapped and assigned to 141 KEGG pathways (S4 Table). The most represented KEGG pathways fell under the ‘metabolism’ category followed by the ‘organismal systems’, ‘environmental information processing’ and ‘genetic information processing’ categories. A large portion of the transcripts was mapped to pathways in the ‘metabolism’ category, including the ‘purine metabolism’ (3488), ‘thiamine metabolism’ (1865) and ‘biosynthesis of antibiotic’ (960) pathways. *Rafflesia* transcripts were also mapped to the ‘T cell receptor signaling pathway’ (239) and ‘Th1 & Th2 cell differentiation’ pathway (203) in the ‘organismal systems’ category, and ‘phosphatidylinositol signaling system’ pathway (219) and ‘mTor signaling pathway’ (34) in the ‘environmental information processing’ category.

### Transcriptome dynamics and co-expression analysis of DEGs

**Fig 4:**
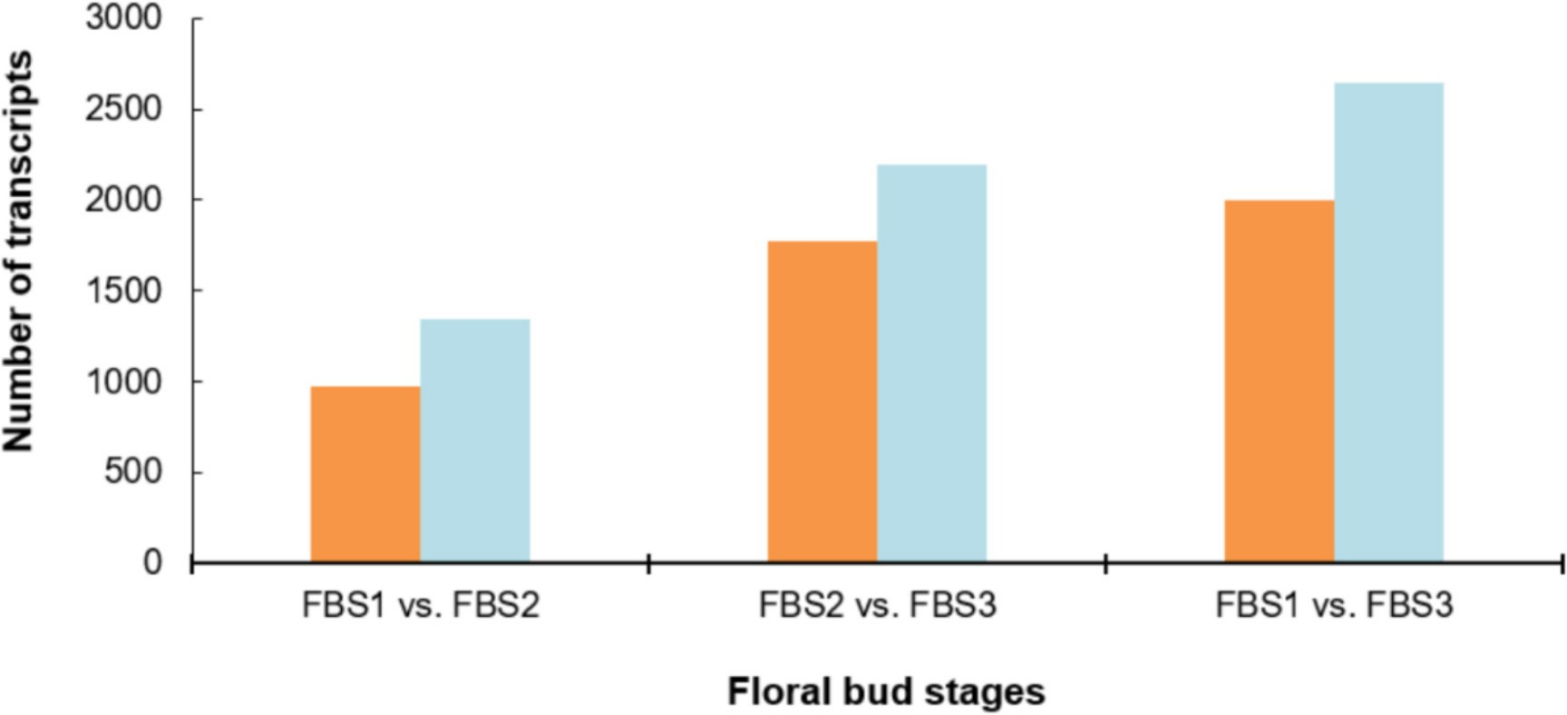
Number of DEGs in different comparisons, including FBS1 vs. FBS2, FBS2 vs. FBS3, and FBS1 vs. FBS3. Significant DEGs were defined by P-value ≤ 0.001 with FDR ≤ 0.05 and |Log2 fold change| > 2. Left bar represents up-regulated transcripts and right bar represents down-regulated transcripts.

We compared the expression levels of each transcript between the three floral bud stages based on pairwise DEG analysis (Fig 4). In the FBS1-FBS2 comparison, 2,312 differentially expressed transcripts were detected, in which 927 transcripts were up-regulated and 1,340 were down-regulated. In the FBS2-FBS3 comparison, 3,961 differentially expressed transcripts were detected in which 1,769 transcripts were up-regulated and 2,192 transcripts were down-regulated. In the FBS1-FBS3 comparison, 4,642 differentially expressed transcripts were identified with 1,999 up-regulated and 2,643 down-regulated.

**Fig 5:**
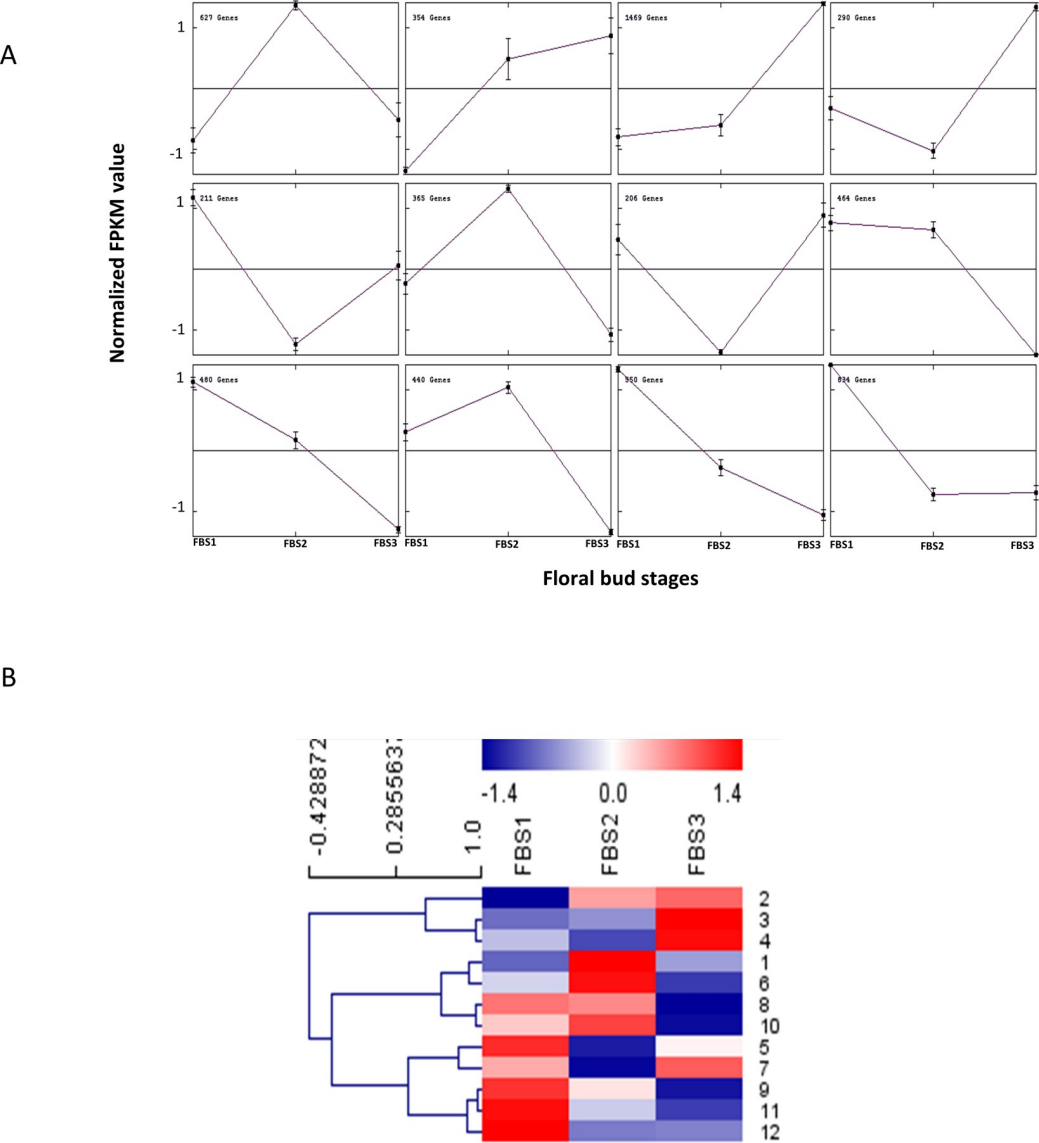
Expression profile analysis of DEGs in *Rafflesia cantleyi* floral bud development. (A) MeV cluster analysis of DEGs from the expression profiles across three floral bud stages (FBS1, FBS2 and FBS3). Main line in each of the 12 cluster graphs indicates the average expression level of transcripts grouped into the same cluster under different floral bud stages. (B) Heatmap for cluster analysis of the DEGs by the K-means method with reference to the scale bar at the top of the figure.

In total, 6,290 DEGs were assigned to 12 clusters by the K-means method (Fig 5A), which reflect the major trends and the key transitional states during the flower development process in *R*. *cantleyi*. Clusters 3 and 8 with 1,469 and 464 transcripts respectively showed opposite pattern whereas clusters 1 and 6 showed the same pattern. Transcripts in clusters 9 and 11 expressed at the earliest floral stages were subsequently down-regulated compared to clusters 2 and 3, which showed up-regulation from FBS1 to FBS3, indicating their different roles in flower development. The DEGs in clusters 5, 8, 9, 11 and 12 were highly expressed in FBS1, while the DEGs showing FBS2 stage-specific expression level were grouped into clusters 1, 6 and 10. DEGs showing FBS3 stage-specific expression were grouped into clusters 2, 3 and 4, which indicate that they may be involved in bud outgrowth. DEGs assigned to clusters 3 and 4 were activated during early flower development when organ primordia were initiated and remain expressed (highly expressed in FBS3) until flowers have reached maturity, suggesting that many of them might play roles in floral organ development. The DEGs in clusters 3 and 4 include zinc finger transcription factor *MIF1* that are involved in mediating the control of Arabidopsis development by multiple hormones including abscisic acid (ABA), auxin, brassinosteroid, cytokinin and gibberellin (GA) [33], as well as genes involved in jasmonate biosynthesis (*ACX1*, *ACX5*, *LOX3*, *LOX4*), jasmonate response (*MYB24*) and auxin response (*IAA4*). This indicates that hormone-signalling pathways play a significant role in *R*. *cantleyi* flower development.

The centroid values of each cluster, which specify the expression pattern, were clustered to compare the relationship between clusters (Fig 5B). Three major groups were identified by K-means method, which showed correlation to three developmental stages. This suggests the association of specific genes cluster with developmental processes that coincide with anatomical and morphological changes of the floral bud.

### Identification of transcription factors related to flower development

Transcription regulators act through the interplay between transcription factors and specific regions of the genome to change the gene expression and causes developmental changes in plants. Some TFs are considered master regulators as their expression affect downstream activation or repression of other TFs. Among TF families, MADS-box [34], MYB [35], ARF [36–37], and AP2 [38] were found to be particularly important during flower and reproductive development in different plant species.

**Fig 6:**
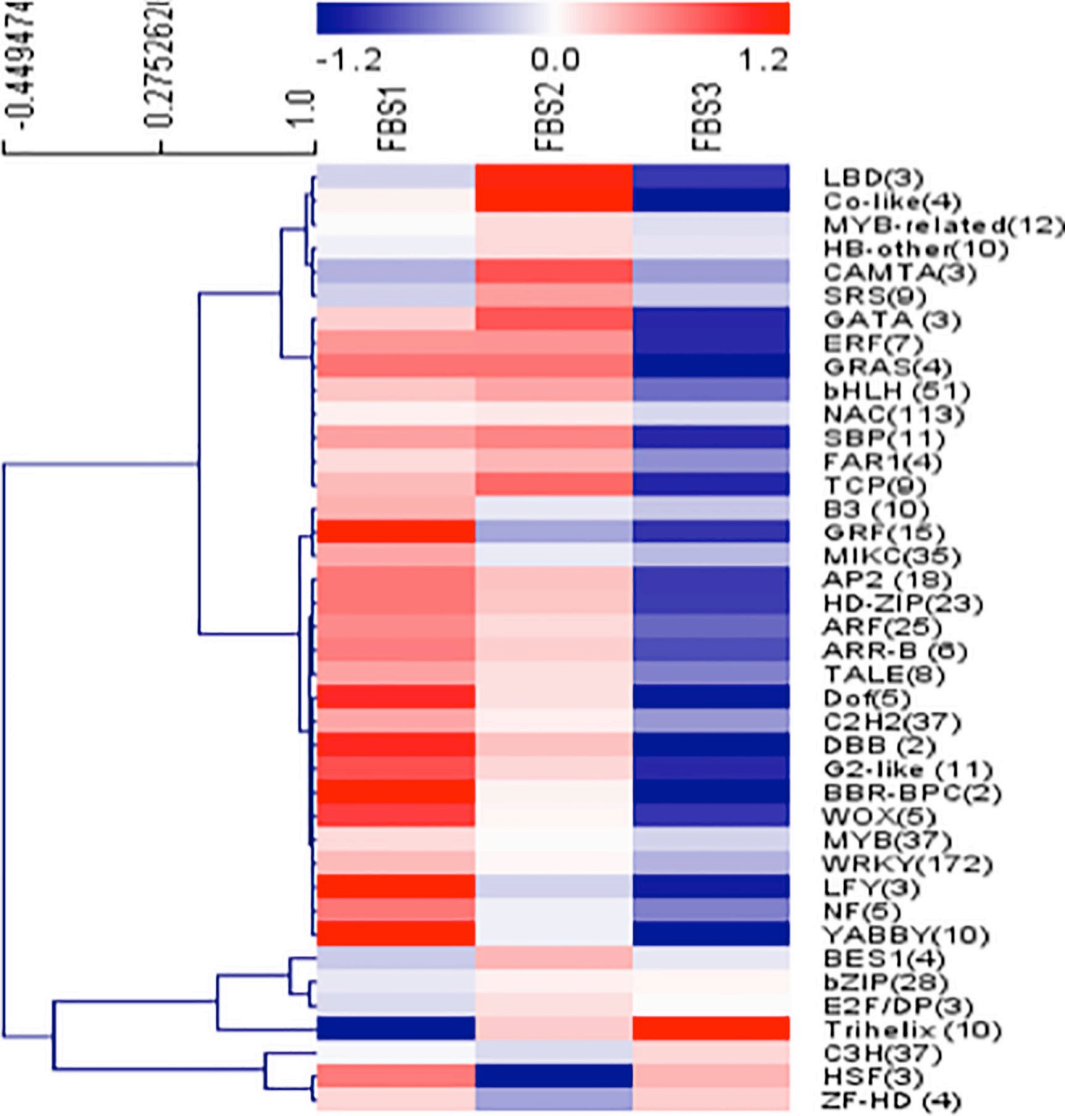
Analysis of transcription factors associated with flower development in *Rafflesia cantleyi*. A heat map depicting the overall trend of the differential expression profiles of the transcription factor during flower development with reference to the scale bar at the top of the figure. The number of DEGs in each transcription factor family is shown in parentheses.

In total, 1,860 TFs from 54 families were identified in floral bud transcriptome from search against PlnTFDB. Among the top 10 abundance during floral bud development, several large transcription factor families include WRKY (279 transcripts), bHLH (173 transcripts), C2H2 (143 transcripts) and NAC (123 transcripts) (S3 Fig). A total of 761 DEGs were annotated as differentially expressed TFs, which were grouped into 40 families (Fig 6). The largest number of differentially expressed TFs comprised of the WRKY, NAC, bHLH, MYB, C2H2, and MIKC families.

Several TF families were significantly up-regulated from FBS1 to FBS3, such as bZIP, E2F/DP and Trihelix in cluster 2 (S3B Fig). In contrast, other TF families including MYB-related, HB-other, BES1 and SRS in cluster 3, and NAC, SBP, bHLH and TCP in cluster 4, were significantly down-regulated after FBS2 (S3 Fig). Besides that, the majority of TFs families such as MYB, WRKY, MIKC, GRF, C2H2, ARF and HD-ZIP in cluster 5 were significantly down-regulated from FBS1 to FBS3 (S3 Fig).

### Identification of transcripts involved in pathways of various flower development-related hormones

**Fig 7:**
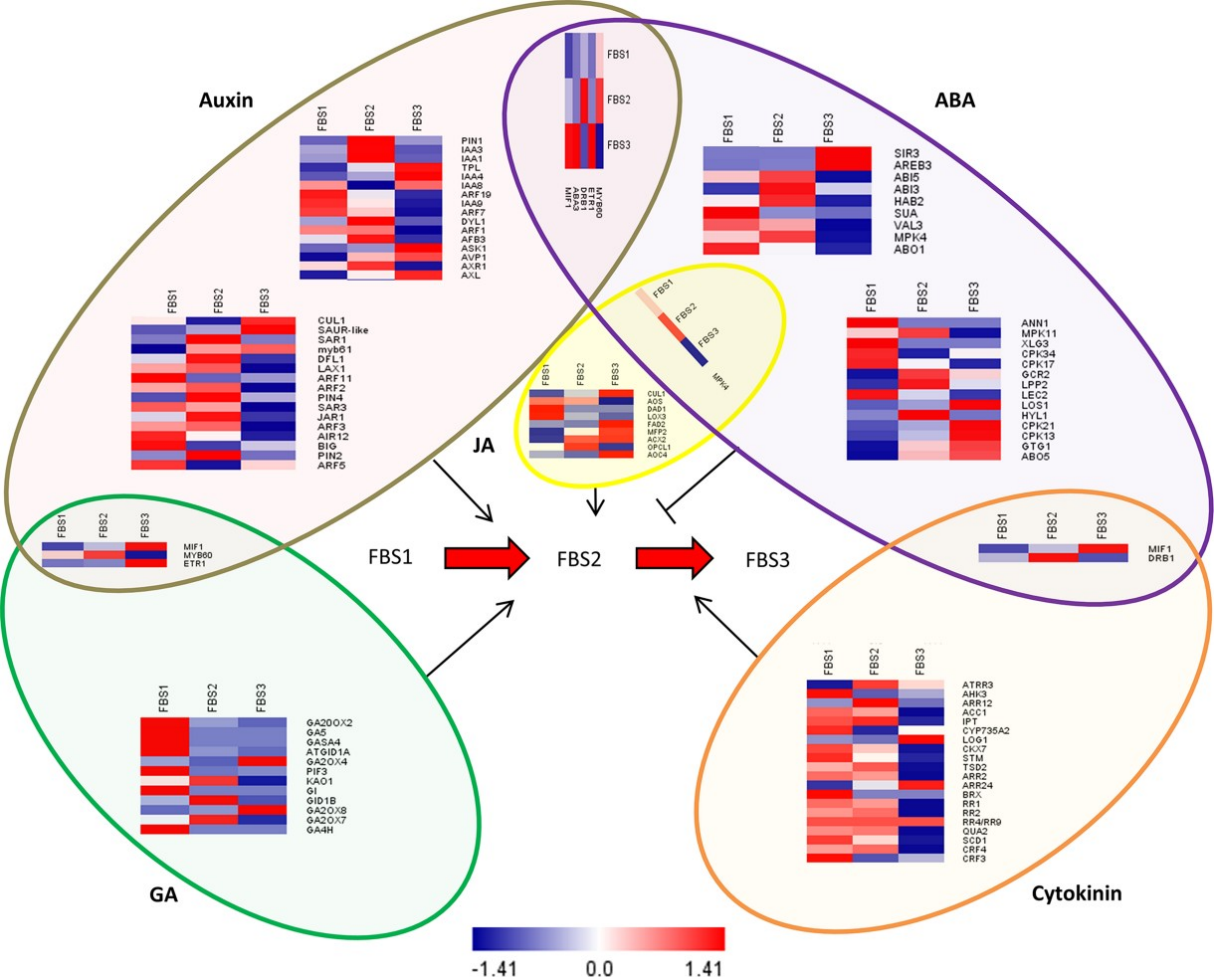
Heat map analysis of various hormones involved in the regulation of flowering process. Log2-transcription FPKM values of each gene correspond to the scale bar at the bottom of the figure. Crosstalk between different hormones during flower development is shown as overlapping borders.

The role of phytohormones including auxin, ABA, cytokinin, GA, and jasmonic acid (JA) in flower development has been well studied [39]. In this study, a total of 37 key regulators in the auxin signalling pathway, 30 key regulators in the ABA signalling pathway, 20 key regulators in the cytokinin signalling pathway, 15 key regulators in the GA signalling pathway, and seven key regulators of JA signalling pathway were identified in our data. Most of these key regulators showed a stage-specific expression pattern. Moreover, five key regulators were identified in both auxin and ABA signalling pathways, three key regulators were identified in both auxin and GA signalling pathways, two key regulators were identified in both ABA and CK signalling pathways, and one key regulator was identified in both ABA and JA signalling pathways. Based on our result, no shared transcript was found between GA and cytokinin signalling pathways during flower development process in *R*. *cantleyi* (Fig 7).

### Verification of gene expression through RT‑qPCR

**Fig 8:**
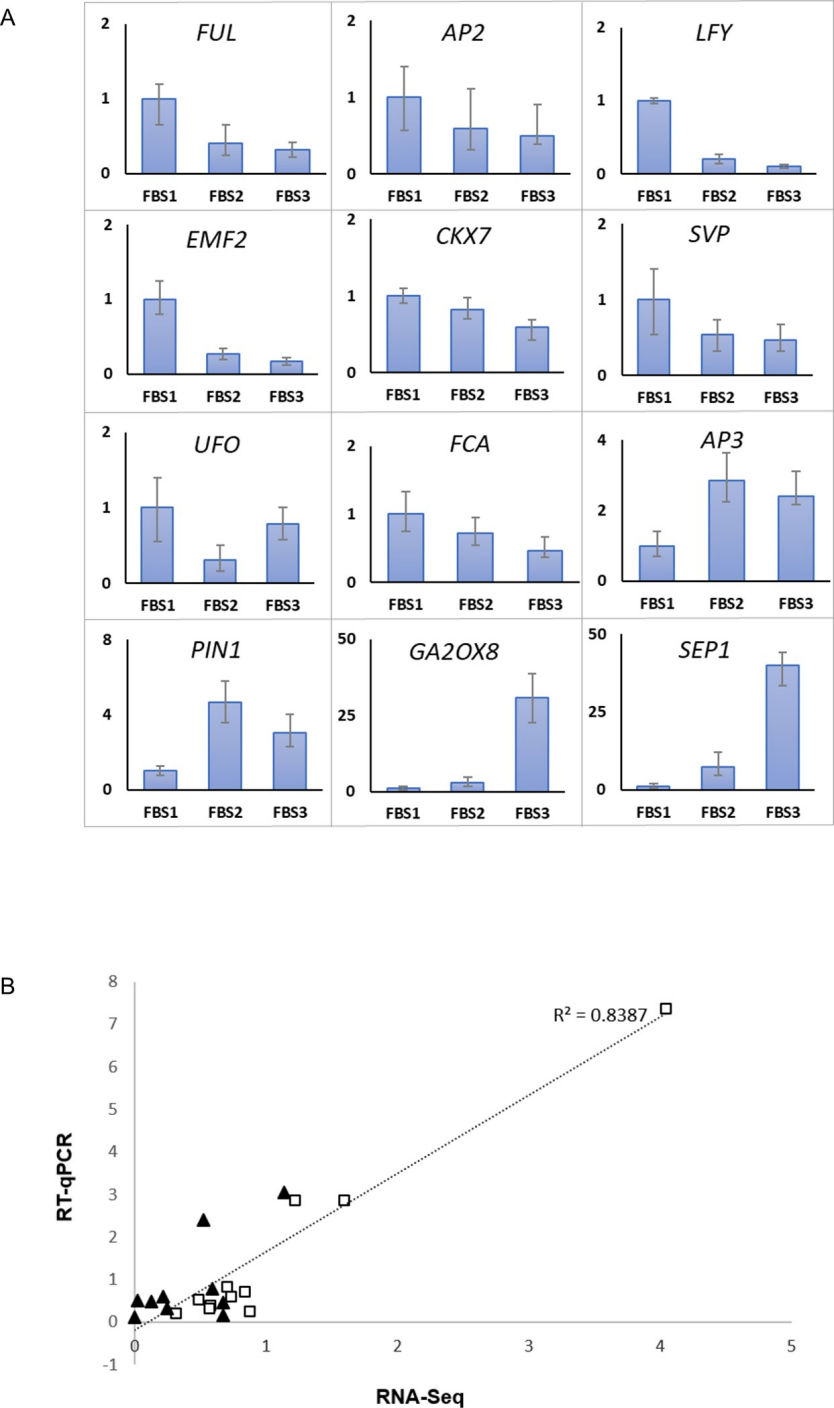
RT-qPCR validation of RNA-seq analysis. (A) Expression of twelve genes related to flower development in different stages of *R*. *cantleyi* floral bud development based on three independent biological replicates. Error bars show ± SD of three technical replicates of the three biological replicates. (B) Scatter plot of fold change (FC) of 12 selected gene expression ratios from RT-qPCR and RNA-Seq data. Squares correspond to FBS1-FBS2 and triangles correspond to FBS1-FBS3 comparison.

To validate the RNA-seq data, we randomly selected 12 transcripts and performed RT-qPCR analysis on samples collected from all three different developmental stages (Fig 8A). RT-qPCR results were highly correlated with RNA-seq data (R^2^ > 0.8) (Fig 8B). This supports that transcript abundance estimation from transcriptomic analysis was reliable.

## Discussion

### *R*. *cantleyi* floral bud functional transcriptome profiling

In plants, morphological and gene expression changes reflect developmental stage transitions. During *R*. *cantleyi* floral bud development, the bud size progressively increased and bracts detached as the buds grow. In the young floral bud, three whorls of sepal, petal and stamen are notable. Floral organs differentiation is apparent at FBS2 and become fully mature at FBS3. Of the assembled transcriptome, only 35% of all transcripts were annotated in *R*. *cantleyi* against the NCBI Nr protein database, which is much fewer than distantly related species such as *V*. *vinifera* (69%) and *T*. *cacao* (56.1%) (Argout *et al*., 2008; da Silva *et al*., 2005). The proportion reported functional annotations to range from 13% to 86% in the literature, depending on the species, the sequencing depth, and the parameters of the BLAST search [40–41]. Apart from technical issues during sequencing, many transcripts without BLAST hit might be caused by biological factors, like fast-evolved genes (having orthologs in other species but so highly divergent that hardly recognised as orthologs), species-specific genes (present in the studied species but absent from the databases), and the perseverance of non-coding fractions mainly from untranslated regions of the transcripts [42]. This indicates that the functions of a large portion of the genes of *R*. *cantleyi* have not yet been identified. It is also noteworthy that scarce genomic resources are available in databases for Rafflesiaceae (*Rafflesia*, *Rhizanthes*, *Sapria*) compared to its close relative, Euphorbiaceae, which Rafflesiaceae believed to be derived from [43]. Given that the genomic and transcriptomic information is limited for *R*. *cantleyi*, the lineage-specific genes associated with the unique floral morphology may not have function annotation, implying potential for novel genes to be discovered from our transcriptome dataset and for further functional studies of this species and its relatives. Interestingly, most transcripts hit to *V*. *vinifera*, which shares the same family (Vitaceae) with the host of *Rafflesia*, *Tetrastigma*. It has been reported that some actively transcribed genes in *Rafflesia* are likely of host origin [44]. This is supported by the identification of large number of homologous *R*. *cantleyi* transcripts to *V*. *vinifera*.

In this study, we performed comparative analysis of gene expression of three floral bud stages. We identified a number of DEGs involved in various biological processes, which are likely to be associated with the regulation of the *R*. *cantleyi* floral bud development. The number of down-regulated genes is larger than up-regulated genes between FBS1 and FBS2, indicating that flower development requires more gene suppression than activation in *R*. *cantleyi* and the onset of flower development was accompanied by the repression of many genes similar to previously reported in Arabidopsis during the incipient of floral primordia [45]. In our study, down-regulated genes between FBS1 and FBS2 include several transcription factor families, such as MIKC, ARF, GRF, MYB, NF, and AP2 (S3B Fig). Members of transcription factor families, including NAC, ARF, NF, MYB, SBP, and HD-ZIP, have been predicted as regulatory targets of miRNAs which are implicated in plant developmental patterning or cell differentiation [46]. Also, it has been suggested that miRNA-transcription factor module regulates lateral organ size and patterning in Arabidopsis [47]. Plant miRNAs are reported to control developmental decisions by down-regulating important developmental transcription factors including AP2-like [48], AP2 [38], MYB and NAC [49]. In our study 30 out of 66 transcripts involved in miRNA production (GO:0035196) were up-regulated between FBS1 and FBS2. Besides, the GO enrichment analyses of FBS1-FBS2 DEGs supported this notion that functional categories including ‘negative regulation of transcripts’ and ‘reproductive system development’ were significantly enriched (S4 Fig).

Furthermore, through comparative gene expression analysis, we found that the transition from FBS1 to FBS2, which reflects the differentiation of floral organs in *R*. *cantleyi* is characterised by a massive gene down-regulation. Unlike our data, the previous study on Arabidopsis incipient floral primordia reported the predominance of gene activation during the differentiation of floral organs [45]. Although there was a significant difference between Arabidopsis and *R*. *cantleyi* in the number and expression pattern of genes with differential expression, it is interesting to note that the same members of TF families were identified in both Arabidopsis and *R*. *cantleyi* including member of bHLH, MADS box, MYB and AP2 families.

Besides that, our analysis showed that there is a higher number of DEGs in FBS2-FBS3 compared to FBS1-FBS2. The higher number of DEGs at the later stage of the flower development was also reported in other plants [50–51], which might indicate that *R*. *cantleyi* bud was going through a basal metabolic activity before entering an active growth stage. Although the number of DEGs in FBS1-FBS2 is less than FBS2-FBS3 and FBS1-FBS3, the remarkable morphological alteration at FBS2 indicates the involvement of complex developmental event with fewer numbers of DEGs. Moreover, the DEGs in FBS2-FBS3 showed greater numbers of genes appeared to be differentially expressed for promoting floral bud maturation. The floral bud outgrowth is associated with various pathways such as starch and sugar biosynthesis, cell cycle regulation [52] and cytokinin signalling pathways [53], which are cooperatively required for organogenesis. The GO enrichment analyses of DEGs supported this concept that functional categories including ‘carbohydrate derivative catabolism’ and ‘cell division’ in FBS1-FBS3, and ‘cytokinin-activated signaling pathway’ in FBS2-FBS3 were significantly enriched (S4 Fig). Besides that, 40% of all DEGs (2,539 transcripts) were grouped into clusters 5, 8, 9 and 11 that showed FBS1-preferential expression (Fig 5), suggesting the significance of gene down-regulation in underlying the stage transition from FBS1 to FBS2 in *R*. *cantleyi* flower development.

### TFs involved in the *R*. *cantleyi* flower development

Previous molecular studies have shown the important roles of TFs in the reproductive development of plants [34]. Amongst the TF families, MYB, bHLH, NAC, WRKY, ARF and GRF are particularly important during flower development in *R*. *cantleyi*. The GRFs form a small transcription family with nine and 12 members in Arabidopsis and *Oryza sativa*, respectively. GRFs consist of two highly conserved regions, the QLQ (Gln, Leu, Gln) and the WRC (Trp, Arg, Cys) domains. Genetic data have shown that GRFs act as transcription activators that are involved in regulating the morphogenesis of leaf and petal [54]. The high abundance of GRF transcripts in actively growing and developing tissue such as immature leaf and flower bud suggests their role in regulating cell proliferation [55]. The significantly high expression of GRFs (16 transcripts) at FBS1 showed these genes might operate similarly in this plant.

The MYB protein family has been identified as a flower developmental regulator [35], which is involved in the regulation of secondary metabolism, control of cellular morphogenesis, regulation of meristem formation, and cell cycle [56]. It has been shown that several MYBs (*MYB21*, *MYB24*, and *MYB57*) are DELLA-responsible GA-response genes that are involved in stamen and pollen maturation [57]. Interestingly, the homologues of *MYB24* and *MYB57* genes were also found in *R*. *cantleyi*. Significantly high expression of MYB genes during the early stage of flower development suggests an important regulatory role of MYBs in *R*. *cantleyi* flower development.

ARFs function to regulate auxin-responsive genes. In Arabidopsis, *ARF3* is involved in regional identity determination by integrating *AG* and *AP2* in floral meristem determinacy [37]. *ARF1* and *ARF2* regulate floral organ abscission and bind directly to the FT promoter to repress floral transition in Arabidopsis [58]. In *R*. *cantleyi*, 15 putative ARF homologues including *ARF1*, *ARF2*, and *ARF3* were highly expressed in FBS1 indicating their importance in the early development of *R*. *cantleyi* floral bud.

The NAC and bHLH families regulate various flower developmental processes. In Arabidopsis, the bHLHs regulate the developmental signalling pathways, including light signalling [59] and flowering time [60]. A total of 51 putative bHLHs homologues were identified as DEGs, which implies their potential role in *R*. *cantleyi* flower development. NACs are known to be involved in embryonic, vegetative and flower development [46]. Several NAC transcription factor genes including *ANAC075*, *SND2* and *NST1* are key regulators of the secondary wall formation in Arabidopsis growth and development [61–62]. In our data, these genes were expressed throughout the bud stages, with significantly up-regulated *NST1* in FBS3, suggesting their role in latter development of *R*. *cantleyi* floral bud.

### The ABC genes in *R*. *cantleyi* flower development

According to the ABC model, A function genes (*AP1* and *AP2*) specify the identity of the sepal, A function genes together with B function genes (*AP3* and *PI*) specify the petals, B function genes together with C function genes (*AG*) specify the stamen, and C function genes specify the carpel [10]. The homologues of ABC model genes were identified in *R*. *cantleyi*. In a previous study, *AP3*, *PI*, *AG* and *SEP1* were detected in *Rafflesia* [14]. In our study, *AP2*, *AP3*, *PI*, *AG* and *SEP1* were identified while *AP2*, *PI*, *AG* and *SEP1* were identified as differentially expressed genes. Interestingly, in the GO enrichment analyses of DEGs, the GO term ‘specification of floral organ identity’, which refers to ABC model genes was only identified in FBS1–BFS2 DEGs cluster and the GO term ‘cell morphogenesis involved in differentiation’ was identified in FBS2–FBS3, which are in very good agreement with morphological observations as well as DEG profiling (S4 Fig). However, the floral chamber of *R*. *cantleyi* is composed of modified organs which are specialised in function and structure, showing that the regulatory mechanism of A, B and C function genes should be further studied in this species. These genes are important candidates for molecular cloning and functional analysis of flower development regulation in *R*. *cantleyi*.

### Hormone-related gene expression in *R*. *cantleyi* flower development

**Fig 9:**
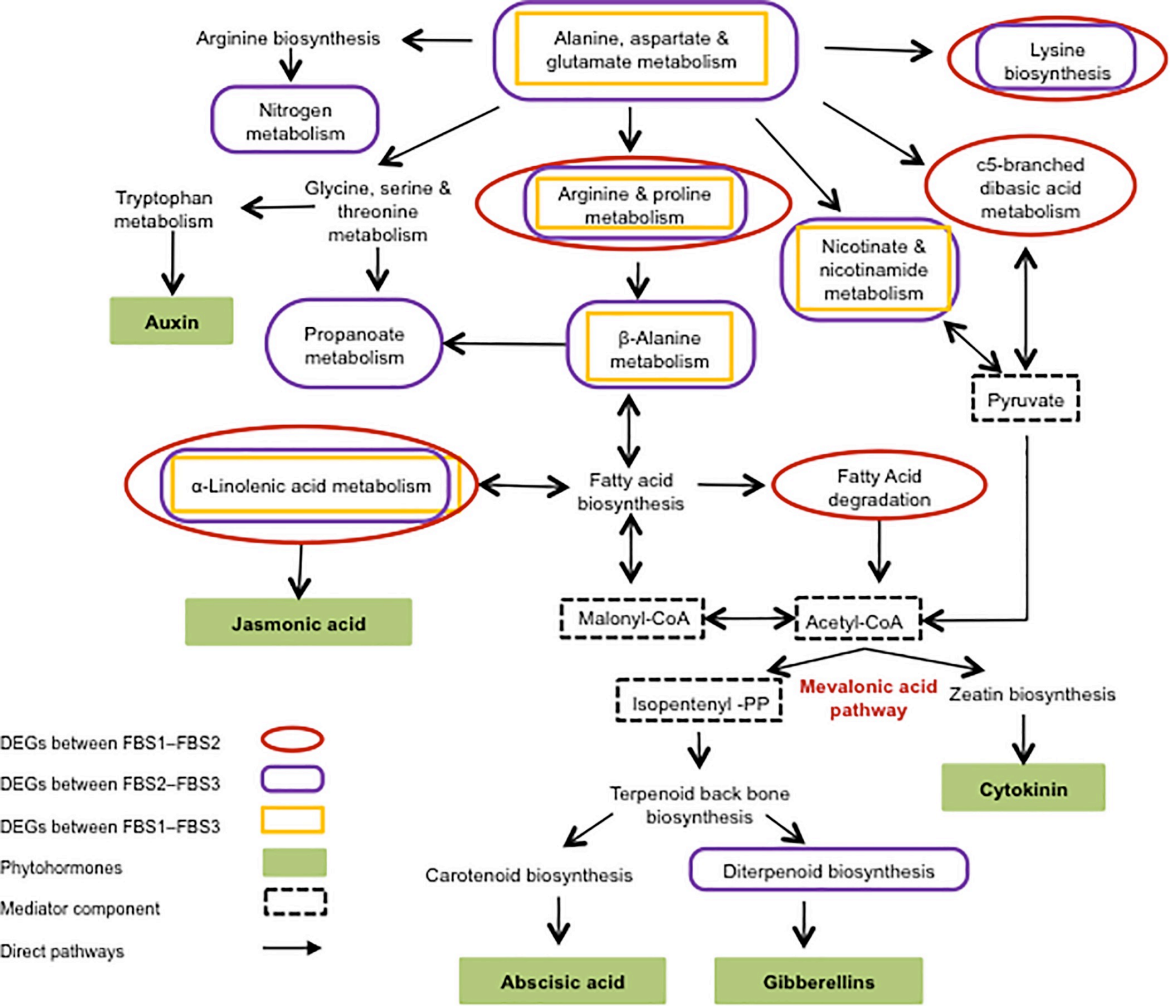
Pathway connection of hormonal signal transduction obtained from GO enrichment analysis of DEGs in *Rafflesia cantleyi*. GO enrichment analysis revealed DEGs involved in various phytohormones metabolism and signal transduction processes.

KEGG pathway enrichment analysis showed that the significantly enriched DEGs were involved in various phytohormones metabolism and signal transduction processes (Fig 9, S5 Table). Also, GO enrichment analysis of DEGs showed that phytohormones might play a role in flower development of *R*. *cantleyi* (S4 Fig).

Auxin plays an essential role in the diverse aspects of flower development including the initiation of floral primordial and floral organ identity [63] as can be seen in Arabidopsis and *Camellia azalea* [64–65]. In *R*. *cantleyi*, a number of auxin-related genes showed significant differential expression during floral bud differentiation. Previous study on *R*. *cantleyi* flower reported that the AUX/IAA and ARF families were constitutively expressed in all flower stages [66]. The homologues of *ARFs* and *AUX-IAA* were found in floral bud, indicating the crucial role of ARF-AUX/IAA regulatory pathway for *R*. *cantleyi* flower development. Among the genes that were differentially expressed, six auxin responsive factor homologues including *ARF1*, *ARF3*, *ARF5*, *ARF7*, *ARF11*, and *ARF19* were highly expressed, whereas homologues of *IAA1*, *IAA3*, *IAA8* and *SAUR-lik*e were lowly expressed during FBS1 suggesting dynamic auxin signalling in *R*. *cantleyi* floral initiation and organogenesis (Fig 7). In the model plant Arabidopsis, *IAA8* was identified as a component of auxin response machinery, interacting with both *ARF6* and *ARF8* to change JA levels, which is involved in flower development [65]. In *R*. *cantleyi*, *IAA8* showed high expression during flower development process, suggesting a putative function of *IAA8* in flower development. *IAA4* showed interactions with *IAA1* and *IAA2* to regulate auxin biosynthesis during carpel development [67]. In our study, *IAA4* was predominantly expressed in FBS3, in contrast to *IAA1*, suggesting their different functions in flower development of *R*. *cantleyi*. Other than that, *PINs*, which are auxin efflux carrier-encoding genes and are important part of a network for auxin distribution throughout the plant, are involved in primordium development and formation of all plant organs [68]. *ARF3* is involved in regional identity determination by integrating *AG* and *AP2* in floral meristem determinacy [37]. *PIN2*, *PIN4* and *ARF3* showed the highest expression during FBS2, suggesting their putative function in organ formation. Thus, auxin and auxin transport may be required for floral meristem determinacy and organ formation in *R*. *cantleyi*.

GA also plays an important role in flower development by regulating floral meristem genes *LFY* and cross-talks with other hormones for their growth control function [69]. Amongst the GA related genes, the GA20 family has been identified in different plant species. In our study, most of the homologues related to GA were highly expressed during the FBS1 except two GA biosynthesis genes, *GA2OX4* and *GA2OX8*. This showed the diversity of regulatory mechanisms in GA biosynthesis during flower development. Also, gibberellin biosynthesis related transcripts (diterpenoid biosynthesis) showed differential genes expression in pre-anthesis stage (Fig 7). This may suggest gibberellin biosynthesis functions to promote late stages of organ development, which is consistent with a previous finding regarding the involvement of GA through repression of *DELLA* protein activity in promoting late stages of petal, stamen and gynoecium development in Arabidopsis [70].

Apart from auxin and GA, cytokinin has also been reported to be involved in flower development by activating MADS-box genes in *Sinapis alba* [71]. In our study, many cytokinin signalling related transcripts and MADS-box genes were identified as DEGs, proposing an involvement of cytokinin-MADS pathway in the regulation of flower development in *R*. *cantleyi*. *SHOOT MERISTEMLESS* (*STM*), a promoter of cytokinin biosynthesis gene through the activation of *ISOPENTYL TRANSFERASE 7* (*IPT7*), is required to prevent stem cells from being incorporated into organ primordia, and thus from differentiating [72], which is in good agreement with its high expression in FBS1. The biosynthesis of cytokinin involves three key enzymes encode by *IPT*, *CYP735A* and *LONELY GUY* (*LOG*). In contrast, cytokinin oxidase (CKX) degrades the active cytokinins, lowering their cellular level [73]. Regardless of decreased *IPT* expression, increased *CYP735A* and *LOG* expression together with decreased CKX expression suggest that there is an increase in cytokinin biosynthesis during floral bud break. Based on GO enrichment analysis of DEGs, the GO term ‘cytokinin-activated signaling pathway’ were identified in FBS2-FBS3 while ‘auxin-activated signaling pathway’ were identified in FBS1-FBS2 cluster, which suggests specific regulators were required for the stage transition in *R*. *cantleyi*, and different developmental pathways were converged for orchestrating the floral bud development (S4 Fig).

The role of ABA is not well understood in the regulation of flower development. The inhibitory effect of ABA on flower development is mediated by *ABSCISIC ACID-INSENSITIVE 5* (*ABI5*) in Arabidopsis [74]. High expression of *ABI5* in FBS2 may result in the inhibition of *R*. *cantleyi* development. In addition, SnRK functions as a positive regulator of ABA signalling pathway, which is directly repressed by PROTEIN PHOSPHATASE 2C (PP2C). PP2C, on the other hand, is inhibited by ABA receptor PYRABACTIN RESISTANCE 1-LIKE (PYL) [75]. *SnRK2*.*6*, *SnRK3*.*4* and *PYL9* showed relatively constant expression level in FBS1 and FBS2 with down-regulation in FBS3 whereas *HIGHLY ABA-INDUCED PP2C GENE 2* (*HAI2*) was significantly up-regulated in FBS3, which might also indicate that ABA functions as an inhibitor in *R*. *cantleyi* flower development.

In addition, arginine is involved in the production of nitric oxide, polyamines and proline, which play crucial roles in regulating developmental processes [76]. Alpha-linolenic acid metabolism is essential for JA biosynthesis. JA occurs organ-specifically in flowers and involves in flower development and fertility [77]. Moreover, enriched C5-branched dibasic acid metabolism and alpha-linolenic acid metabolism pathways were shown to be associated with cell fate transition [78]. Taken together, KEGG enrichment of DEGs related to arginine and proline metabolism, C5-branched dibasic acid, and alpha-linolenic acid metabolism pathways suggests the importance of JA in *R*. *cantleyi* flowering (Fig 9).

## Conclusions

In this study, the analysis of transcriptional changes during flower development in *R*. *cantleyi* showed that initial flower development involved suppression on various genes with higher number of DEGs identified approaching maturation. Down-regulated genes related to various TF families and highly expressed hormone-related genes further suggested their importance in *R*. *cantleyi* flower development. The identification of these DEGs provides opportunity for further studies to elucidate their functions and contribute towards the understanding of flower development in *Rafflesia* and other plant species.

## Supporting information

S1 Fig Annotation of *Rafflesia cantleyi* transcripts. (PDF)

S2 Fig Percentage of second level COG terms of *Rafflesia cantleyi* transcriptome. (PDF)

S3 Fig Abundance analysis and cluster analysis of transcription factors in *Rafflesia cantleyi* transcriptome. (PDF)

S4 Fig Scatterplot of enriched GO terms for DEGs using semantic clustering (REViGO). (PDF)

S1 Table Primer sequences of reference genes and 12 selected genes for RT-qPCR analysis. (PDF)

S2 Table Functional annotation of *Rafflesia cantleyi* transcripts against Nr, Swiss-Prot, TAIR, PFAM, GO, COG and KEGG databases. (PDF)

S3 Table GO classifications of transcripts. (PDF)

S4 Table KEGG classifications of transcripts. (PDF)

S5 Table KEGG pathways enrichment analysis of DEGs. (PDF)

## References

[1] Adam JH, Mohamed R, Juhari MAA, Ariff NNFN, Wan KL. *Rafflesia sharifah-hapsahiae* (Rafflesiaceae), a new species from Peninsular Malaysia. Turkish Journal of Botany. 2013;37: 1038–1044.

[2] Adam JH, Juhari MAA, Mohamed R, Wahan NAA, Arshad S, Kamruzaman MP, et al *Rafflesia tuanku halimii* n. (Rafflesiaceae), a new species from peninsular Malaysia. Sains Malaysiana. 2016;45: 1589–1595.

[3] Nais J, Rafflesia of the World, Sabah Parks Trustees, Kota Kinabalu 2001.

[4] Davis CC, Endress PK, Baum DA. The evolution of floral gigantism. Current Opinion in Plant Biology. 2008;11: 49–57. 10.1016/j.pbi.2007.11.003 18207449

[5] Meijer W. Rafflesiaceae. Flora Malesiana. 1997;13: 1–42.

[6] Srikanth A, Schmid M. Regulation of flowering time: all roads lead to Rome. Cellular and Molecular Life Sciences. 2011;68: 2013–2037. 10.1007/s00018-011-0673-y 21611891 PMC11115107

[7] Komeda Y. Genetic regulation of time to flower in *Arabidopsis thaliana*. Annual Review of Plant Biology. 2004;55: 521–535. 10.1146/annurev.arplant.55.031903.141644 15377230

[8] Causier B, Schwarz-Sommer Z, Davies B. Floral organ identity: 20 years of ABCs. Seminars in Cell & Developmental Biology. 2010;21: 73–79. 19883777 10.1016/j.semcdb.2009.10.005

[9] Stewart D, Graciet E, Wellmer F. Molecular and regulatory mechanisms controlling floral organ development. The FEBS Journal. 2016;283: 1823–1830. 10.1111/febs.13640 26725470

[10] Huijser P, Schmid M. The control of developmental phase transitions in plants. Development. 2011;138: 4117–4129. 10.1242/dev.063511 21896627

[11] Wee SL, Tan SB, Jürgens A. Pollinator specialization in the enigmatic *Rafflesia cantleyi*: A true carrion flower with species-specific and sex-biased blow fly pollinators. Phytochemistry. 2018;153: 120–128. 10.1016/j.phytochem.2018.06.005 29906658

[12] Ng SM, Lee XW, Mat-Isa MN, Aizat-Juhari MA, Adam JH, Mohamed R, et al Comparative analysis of nucleus-encoded plastid-targeting proteins in Rafflesia cantleyi against photosynthetic and non-photosynthetic representatives reveals orthologous systems with potentially divergent functions. Scientific Reports. 2018;8: 17258 10.1038/s41598-018-35173-1 30467394 PMC6250676

[13] Lee XW, Mat-Isa MN, Mohd-Elias NA, Aizat-Juhari MA, Goh HH, Dear PH, et al Perigone lobe transcriptome analysis provides insights into *Rafflesia cantleyi* flower development. PLoS One. 2016;11: e0167958 10.1371/journal.pone.0167958 27977777 PMC5158018

[14] Nikolov LA, Endress PK, Sugumaran M, Sasirat S, Vessabutr S, Kramer EM, et al Developmental origins of the world’s largest flowers, Rafflesiaceae. Proceedings of the National Academy of Sciences. 2013;110: 18578–18583. 10.1073/pnas.1310356110 PMC3831985 24167265

[15] Amini S, Alias H, Aizat-Juhari MA, Mat-Isa MN, Adam JH, Goh HH, et al RNA-seq data from different developmental stages of *Rafflesia cantleyi* floral buds. Genomics Data. 2017;14: 5–6. 10.1016/j.gdata.2017.07.008 28761813 PMC5524293

[16] Bolger AM, Lohse M, Usadel B. Trimmomatic: a flexible trimmer for Illumina sequence data. Bioinformatics. 2014;30: 2114–2120. 10.1093/bioinformatics/btu170 24695404 PMC4103590

[17] Grabherr MG, Haas BJ, Yassour M, Levin JZ, Thompson DA, Amit I, et al Full-length transcriptome assembly from RNA-Seq data without a reference genome. Nature Biotechnology. 2011;29: 644 10.1038/nbt.1883 21572440 PMC3571712

[18] Wang Y, Pan Y, Liu Z, Zhu X, Zhai L, Xu L, et al *De novo* transcriptome sequencing of radish (*Raphanus sativus* L.) and analysis of major genes involved in glucosinolate metabolism. BMC Genomics. 2013;14: 836 10.1186/1471-2164-14-836 24279309 PMC4046679

[19] Conesa A, Götz S, García-Gómez JM, Terol J, Talón M, Robles M. Blast2GO: a universal tool for annotation, visualization and analysis in functional genomics research. Bioinformatics. 2005;21: 3674–3676. 10.1093/bioinformatics/bti610 16081474

[20] Ye J, Fang L, Zheng H, Zhang Y, Chen J, Zhang Z, et al WEGO: a web tool for plotting GO annotations. Nucleic Acids Research. 2006;34: W293–W297. 10.1093/nar/gkl031 16845012 PMC1538768

[21] Ogata H, Goto S, Sato K, Fujibuchi W, Bono H, Kanehisa M. KEGG: Kyoto encyclopedia of genes and genomes. Nucleic Acids Research. 1999;27: 29–34. 10.1093/nar/27.1.29 9847135 PMC148090

[22] Finn RD, Bateman A, Clements J, Coggill P, Eberhardt RY, Eddy SR, et al Pfam: the protein families database. Nucleic Acids Research. 2013;42: D222–D230. 10.1093/nar/gkt1223 24288371 PMC3965110

[23] Consortium GO. The Gene Ontology (GO) database and informatics resource. Nucleic Acids Research. 2004;32: D258–D261. 10.1093/nar/gkh036 14681407 PMC308770

[24] Pérez-Rodríguez P, Riano-Pachon DM, Corrêa LGG, Rensing SA, Kersten B, Mueller-Roeber B. PlnTFDB: updated content and new features of the plant transcription factor database. Nucleic Acids Research. 2009;38: D822–D827. 10.1093/nar/gkp805 19858103 PMC2808933

[25] Li B, Dewey CN. RSEM: accurate transcript quantification from RNA-Seq data with or without a reference genome. BMC Bioinformatics. 2011;12: 323 10.1186/1471-2105-12-323 21816040 PMC3163565

[26] Robinson MD, McCarthy DJ, Smyth GK. edgeR: a Bioconductor package for differential expression analysis of digital gene expression data. Bioinformatics. 2010;26: 139–140. 10.1093/bioinformatics/btp616 19910308 PMC2796818

[27] Xie C, Mao X, Huang J, Ding Y, Wu J, Dong S, et al KOBAS 2.0: a web server for annotation and identification of enriched pathways and diseases. Nucleic Acids Research. 2011;39: W316–W322. 10.1093/nar/gkr483 21715386 PMC3125809

[28] Supek F, Bošnjak M, Škunca N, Šmuc T. REVIGO summarizes and visualizes long lists of gene ontology terms. PLoS One. 2011;6: e21800 10.1371/journal.pone.0021800 21789182 PMC3138752

[29] Doyle JJ. A rapid DNA isolation procedure for small quantities of fresh leaf tissue. Phytochemical Bulletin. 1987;19: 11–15.

[30] Japelaghi RH, Haddad R, Garoosi GA. Rapid and efficient isolation of high quality nucleic acids from plant tissues rich in polyphenols and polysaccharides. Molecular Biotechnology. 2011;49: 129–137. 10.1007/s12033-011-9384-8 21302150

[31] Reid KE, Olsson N, Schlosser J, Peng F, Lund ST. An optimized grapevine RNA isolation procedure and statistical determination of reference genes for real-time RT-PCR during berry development. BMC Plant Biology. 2006;6: 27 10.1186/1471-2229-6-27 17105665 PMC1654153

[32] Nikolov LA, Staedler YM, Manickam S, Schönenberger J, Endress PK, Kramer EM, et al Floral structure and development in Rafflesiaceae with emphasis on their exceptional gynoecia. American Journal of Botany. 2014;101: 225–243. 10.3732/ajb.1400009 24509798

[33] Hu W, Ma H. Characterization of a novel putative zinc finger gene *MIF1*: involvement in multiple hormonal regulation of Arabidopsis development. The Plant Journal. 2006;45: 399–422. 10.1111/j.1365-313X.2005.02626.x 16412086

[34] Smaczniak C, Immink RG, Muiño JM, Blanvillain R, Busscher M, Busscher-Lange J, et al Characterization of MADS-domain transcription factor complexes in Arabidopsis flower development. Proceedings of the National Academy of Sciences. 2012;109: 1560–1565. 10.1073/pnas.1112871109 PMC3277181 22238427

[35] Vimolmangkang S, Han Y, Wei G, Korban SS. An apple MYB transcription factor, MdMYB3, is involved in regulation of anthocyanin biosynthesis and flower development. BMC Plant Biology. 2013;13: 176 10.1186/1471-2229-13-176 24199943 PMC3833268

[36] Ellis CM, Nagpal P, Young JC, Hagen G, Guilfoyle TJ, Reed JW. AUXIN RESPONSE FACTOR1 and AUXIN RESPONSE FACTOR2 regulate senescence and floral organ abscission in *Arabidopsis thalian*. Development. 2005;132: 4563–4574. 10.1242/dev.02012 16176952

[37] Liu X, Dinh TT, Li D, Shi B, Li Y, Cao X, et al AUXIN RESPONSE FACTOR 3 integrates the functions of AGAMOUS and APETALA 2 in floral meristem determinacy. The Plant Journal. 2014;80: 629–641. 10.1111/tpj.12658 25187180 PMC4215321

[38] Chen X. A microRNA as a translational repressor of APETALA2 in Arabidopsis flower development. Science. 2004;303: 2022–2025. 10.1126/science.1088060 12893888 PMC5127708

[39] Davis SJ. Integrating hormones into the floral‐transition pathway of *Arabidopsis thaliana*. Plant, Cell & Environment. 2009;32: 1201–1210. 10.1111/j.1365-3040.2009.01968.x 19302104

[40] Ness RW, Siol M, Barrett SC. *De novo* sequence assembly and characterization of the floral transcriptome in cross-and self-fertilizing plants. BMC Genomics. 2011;12: 298 10.1186/1471-2164-12-298 21649902 PMC3128866

[41] Parchman TL, Geist KS, Grahnen JA, Benkman CW, Buerkle CA. Transcriptome sequencing in an ecologically important tree species: assembly, annotation, and marker discovery. BMC Genomics. 2010;11: 180 10.1186/1471-2164-11-180 20233449 PMC2851599

[42] Logacheva MD, Kasianov AS, Vinogradov DV, Samigullin TH, Gelfand MS, Makeev VJ, et al *De novo* sequencing and characterization of floral transcriptome in two species of buckwheat (Fagopyrum). BMC Genomics. 2011;12: 30 10.1186/1471-2164-12-30 21232141 PMC3027159

[43] Davis CC, Latvis M, Nickrent DL, Wurdack KJ, Baum DA. Floral gigantism in Rafflesiaceae. Science. 2007;315: 1812–1812. 10.1126/science.1135260 17218493

[44] Xi Z, Bradley RK, Wurdack KJ, Wong K, Sugumaran M, Bomblies K, et al Horizontal transfer of expressed genes in a parasitic flowering plant. BMC Genomics. 2012;13: 227 10.1186/1471-2164-13-227 22681756 PMC3460754

[45] Wellmer F, Alves-Ferreira M, Dubois A, Riechmann JL, Meyerowitz EM. Genome-wide analysis of gene expression during early Arabidopsis flower development. PLoS Genetics. 2006;2: e117 10.1371/journal.pgen.0020117 16789830 PMC1523247

[46] Mallory AC, Dugas DV, Bartel DP, Bartel B. MicroRNA regulation of NAC-domain targets is required for proper formation and separation of adjacent embryonic, vegetative, and floral organs. Current Biology. 2004;14: 1035–1046. 10.1016/j.cub.2004.06.022 15202996

[47] Larue CT, Wen J, Walker JC. A microRNA–transcription factor module regulates lateral organ size and patterning in Arabidopsis. The Plant Journal. 2009;58: 450–463. 10.1111/j.1365-313X.2009.03796.x 19154203

[48] Aukerman MJ, Sakai H. Regulation of flowering time and floral organ identity by a microRNA and its APETALA2-like target genes. The Plant Cell. 2003;15: 2730–2741. 10.1105/tpc.016238 14555699 PMC280575

[49] Schwab R, Palatnik JF, Riester M, Schommer C, Schmid M, Weigel D. Specific effects of microRNAs on the plant transcriptome. Developmental Cell. 2005;8: 517–527. 10.1016/j.devcel.2005.01.018 15809034

[50] Huang YJ, Liu LL, Huang JQ, Wang ZJ, Chen FF, Zhang QX, et al Use of transcriptome sequencing to understand the pistillate flowering in hickory (*Carya cathayensis* Sarg.). BMC Genomics. 2013;14: 691 10.1186/1471-2164-14-691 24106755 PMC3853572

[51] Liu K, Feng S, Pan Y, Zhong J, Chen Y, Yuan C, Li H. Transcriptome analysis and identification of genes associated with floral transition and flower development in sugar apple (*Annona squamosa* L.). Frontiers in Plant Science. 2016;7: 1695 10.3389/fpls.2016.01695 27881993 PMC5101194

[52] Kayal WE, Allen CC, Ju CJT, Adams E, King-Joned S, Zaharia LI, et al Molecular events of apical bud formation in white spruce. *Picea glauca*. Plant, Cell & Environment. 2011;34: 480–500. 10.1111/j.1365-3040.2010.02257.x 21118421

[53] Nordström A, Tarkowski P, Tarkowska D, Norbaek R, Åstot C, Dolezal K, et al Auxin regulation of cytokinin biosynthesis in *Arabidopsis thaliana*: a factor of potential importance for auxin–cytokinin-regulated development. Proceedings of the National Academy of Sciences. 2004;101: 8039–8044. 10.1073/pnas.0402504101 PMC419553 15146070

[54] Horiguchi G, Kim GT, Tsukaya H. The transcription factor AtGRF5 and the transcription coactivator AN3 regulate cell proliferation in leaf primordia of *Arabidopsis thaliana*. The Plant Journal. 2005;43: 68–78. 10.1111/j.1365-313X.2005.02429.x 15960617

[55] Kim JH, Choi D, Kende H. The AtGRF family of putative transcription factors is involved in leaf and cotyledon growth in Arabidopsis. The Plant Journal. 2003;36: 94–104. 10.1046/j.1365-313x.2003.01862.x 12974814

[56] Jin H, Martin C. Multifunctionality and diversity within the plant MYB-gene family. Plant Molecular Biology. 1999;41: 577–585. 10.1023/a:1006319732410 10645718

[57] Cheng H, Song S, Xiao L, Soo HM, Cheng Z, Xie D, et al Gibberellin acts through jasmonate to control the expression of MYB21, MYB24, and MYB57 to promote stamen filament growth in Arabidopsis. PLoS Genetics. 2009;5: e1000440 10.1371/journal.pgen.1000440 19325888 PMC2654962

[58] Gu X, Wang Y, He Y. Photoperiodic regulation of flowering time through periodic histone deacetylation of the florigen gene FT. PLoS Biology. 2013;11: e1001649 10.1371/journal.pbio.1001649 24019760 PMC3760768

[59] Leivar P, Tepperman JM, Cohn MM, Monte E, Al-Sady B, Erickson E, et al Dynamic antagonism between phytochromes and PIF family basic helix-loop-helix factors induces selective reciprocal responses to light and shade in a rapidly responsive transcriptional network in Arabidopsis. The Plant Cell. 2012;24: 1398–1419. 10.1105/tpc.112.095711 22517317 PMC3398554

[60] Ito S, Song YH, Josephson-Day AR, Miller RJ, Breton G, Olmstead RG, et al FLOWERING BHLH transcriptional activators control expression of the photoperiodic flowering regulator CONSTANS in Arabidopsis. Proceedings of the National Academy of Sciences U.S.A. 2012;109: 3582–3587. 10.1073/pnas.1118876109 PMC3295255 22334645

[61] Hussey SG, Mizrachi E, Spokevicius AV, Bossinger G, Berger DK, Myburg AA. SND2, a NAC transcription factor gene, regulates genes involved in secondary cell wall development in Arabidopsis fibres and increases fibre cell area in *Eucalyptus*. BMC Plant Biology. 2011;11: 173 10.1186/1471-2229-11-173 22133261 PMC3289092

[62] Sakamoto S, Mitsuda N. Reconstitution of a secondary cell wall in a secondary cell wall-deficient Arabidopsis mutant. Plant and Cell Physiology. 2014;56: 299–310. 10.1093/pcp/pcu208 25535195 PMC4323883

[63] Alabadí D, Blázquez MA, Carbonell J, Ferrándiz C, Pérez-Amador MA. Instructive roles for hormones in plant development. International Journal of Developmental Biology. 2009;53: 1597 10.1387/ijdb.072423da 19247940

[64] Fan Z, Li J, Li X, Wu B, Wang J, Liu Z, et al Genome-wide transcriptome profiling provides insights into floral bud development of summer-flowering *Camellia azalea*. Scientific Reports. 2015;5: 9729 10.1038/srep09729 25978548 PMC4432871

[65] Wang J, Yan DW, Yuan TT, Gao X, Lu YT. A gain-of-function mutation in IAA8 alters Arabidopsis floral organ development by change of jasmonic acid level. Plant Molecular Biology. 2013;82: 71–83. 10.1007/s11103-013-0039-y 23483289

[66] Elias NAM, Goh HH, Isa NM, Wan KL. Identification of ARF and AUX/IAA gene families in *Rafflesia cantleyi*. AIP Conference Proceedings. 2016: 020017.

[67] Kaufmann K, Muino JM, Jauregui R, Airoldi CA, Smaczniak C, Krajewski P, et al Target genes of the MADS transcription factor SEPALLATA3: integration of developmental and hormonal pathways in the Arabidopsis flower. PLoS Biology. 7 2009: e1000090 10.1371/journal.pbio.1000090 19385720 PMC2671559

[68] Benková E, Michniewicz M, Sauer M, Teichmann T, Seifertová D, Jürgens G, et al Local, efflux-dependent auxin gradients as a common module for plant organ formation. Cell. 2003;115: 591–602. 10.1016/s0092-8674(03)00924-3 14651850

[69] Ribeiro DM, Araújo WL, Fernie AR, Schippers JH, Mueller-Roeber B. Translatome and metabolome effects triggered by gibberellins during rosette growth in Arabidopsis. Journal of Experimental Botany. 2012;63: 2769–2786. 10.1093/jxb/err463 22291129 PMC3346235

[70] Goto N, Pharis RP. Role of gibberellins in the development of floral organs of the gibberellin-deficient mutant, ga1-1, of *Arabidopsis thaliana*. Canadian Journal of Botany. 1999;77: 944–954.

[71] Bonhomme F, Kurz B, Melzer S, Bernier G, Jacqmard A. Cytokinin and gibberellin activate *SaMADS A*, a gene apparently involved in regulation of the floral transition in *Sinapis alba*. The Plant Journal. 2000;24: 103–111. 10.1046/j.1365-313x.2000.00859.x 11029708

[72] Clark SE, Jacobsen SE, Levin JZ, Meyerowitz EM. The CLAVATA and SHOOT MERISTEMLESS loci competitively regulate meristem activity in Arabidopsis. Development. 1996;122: 1567–1575. 8625843 10.1242/dev.122.5.1567

[73] Yamburenko MV, Kieber JJ, Schaller GE. Dynamic patterns of expression for genes regulating cytokinin metabolism and signaling during rice inflorescence development. PLoS One. 2017;12: e0176060 10.1371/journal.pone.0176060 28419168 PMC5395194

[74] Wang Y, Li L, Ye T, Lu Y, Chen X, Wu Y. The inhibitory effect of ABA on floral transition is mediated by ABI5 in Arabidopsis. Journal of Experimental Botany. 2013;64: 675–684. 10.1093/jxb/ers361 23307919 PMC3542054

[75] Cutler SR, Rodriguez PL, Finkelstein RR, Abrams SR. Abscisic acid: emergence of a core signaling network. Annual Review of Plant Biology. 2010;61: 651–679. 10.1146/annurev-arplant-042809-112122 20192755

[76] Winter G, Todd CD, Trovato M, Forlani G, Funck D. Physiological implications of arginine metabolism in plants. Frontiers in Plant Science. 2015;6: 534 10.3389/fpls.2015.00534 26284079 PMC4520006

[77] Wasternack C, Forner S, Strnad M, Hause B. Jasmonates in flower and seed development. Biochimie. 2013;95: 79–85. 10.1016/j.biochi.2012.06.005 22705387

[78] Xiao L, Zhang L, Yang G, Zhu H, He Y. Transcriptome of protoplasts reprogrammed into stem cells in *Physcomitrella patens*. PLoS One. 2012;7: e35961 10.1371/journal.pone.0035961 22545152 PMC3335808

