# Supplementary material for: Transcriptome landscape of Rafflesia cantleyi floral buds reveals insights into the roles of transcription factors and phytohormones in flower development
Source: PLoS One. 2019 Dec 18;14(12):e0226338. doi: 10.1371/journal.pone.0226338 (PMC6919626; doi:10.1371/journal.pone.0226338)
Supplement: S1 Fig — (PDF) [file pone.0226338.s001.pdf]

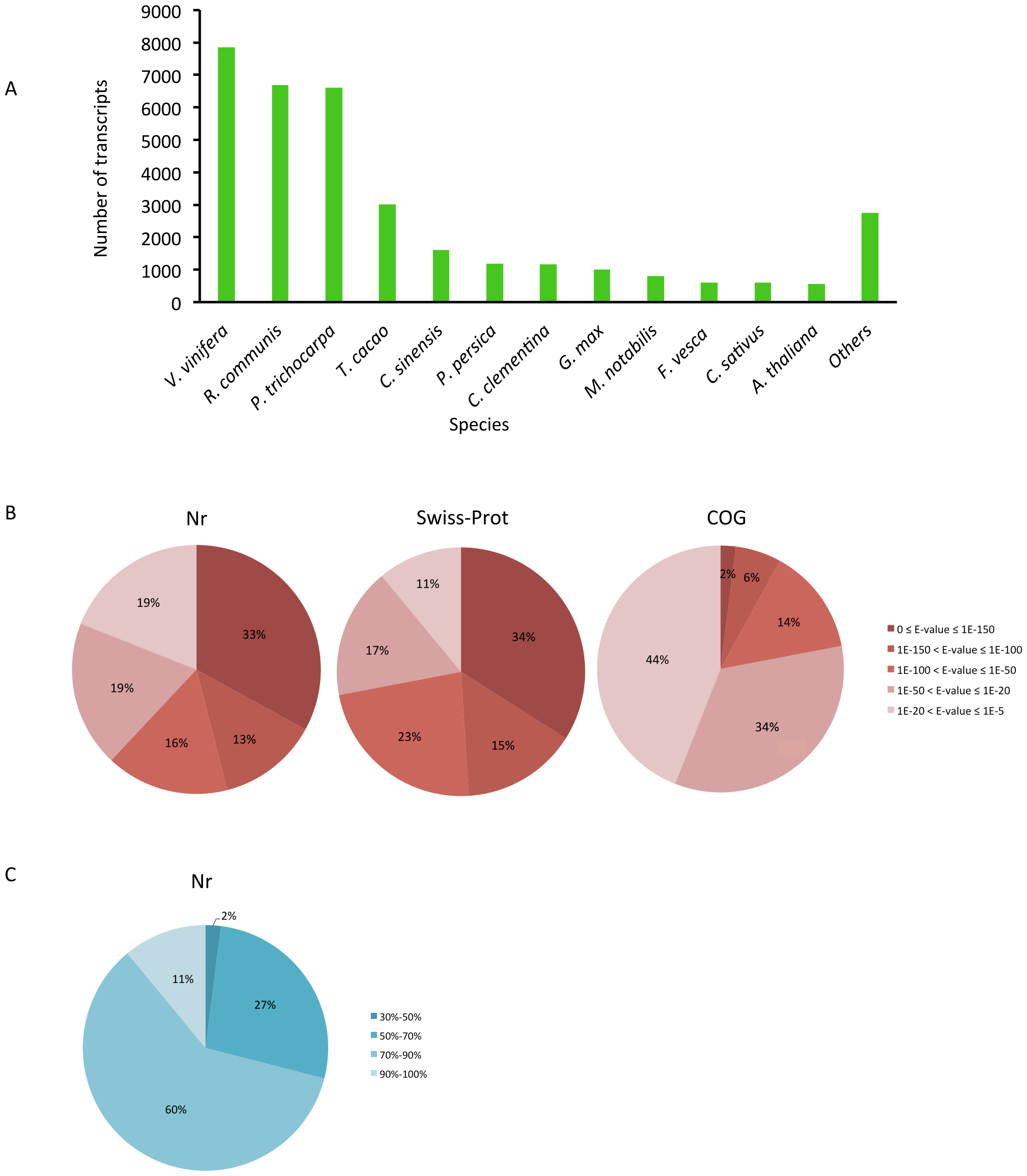

Figure S1. Annotation of *Rafflesia cantleyi* transcripts. (A) Identification of the transcripts of other plant species homologous to the annotated *R. cantleyi* transcripts. (B) Distribution of E-values of BLASTX hits from Nr, Swiss-Prot, and COG database. (C) Distribution of similarity values of top Nr BLASTX hits.
