# Supplementary material for: Transcriptome landscape of Rafflesia cantleyi floral buds reveals insights into the roles of transcription factors and phytohormones in flower development
Source: PLoS One. 2019 Dec 18;14(12):e0226338. doi: 10.1371/journal.pone.0226338 (PMC6919626; doi:10.1371/journal.pone.0226338)
Supplement: S2 Fig — (PDF) [file pone.0226338.s002.pdf]

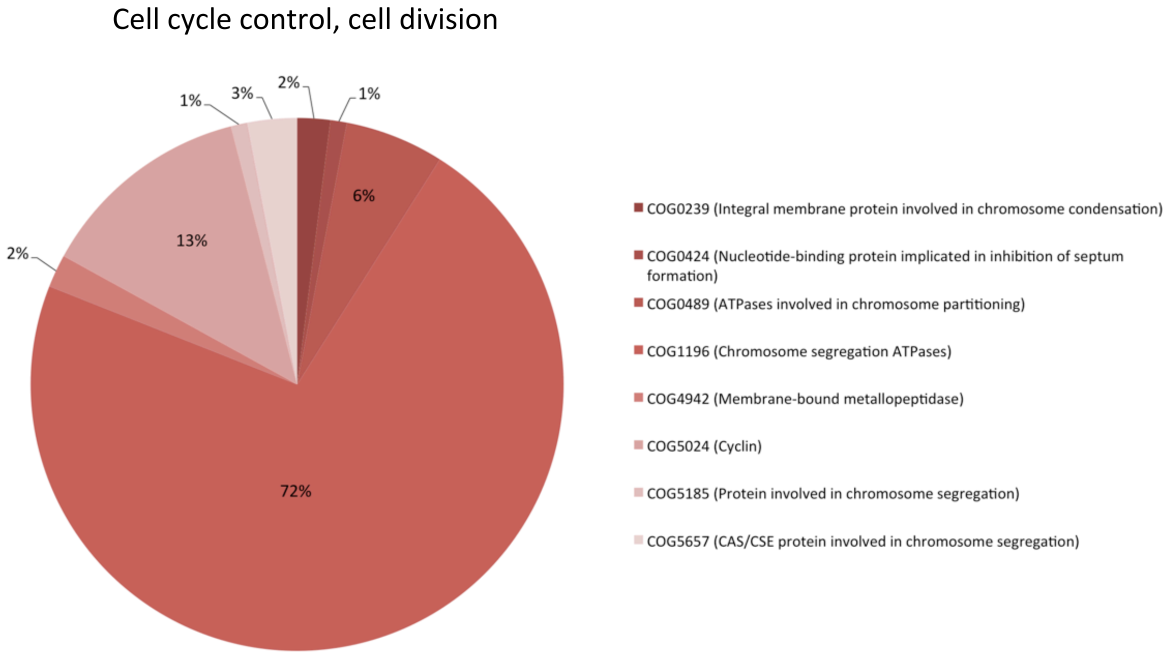

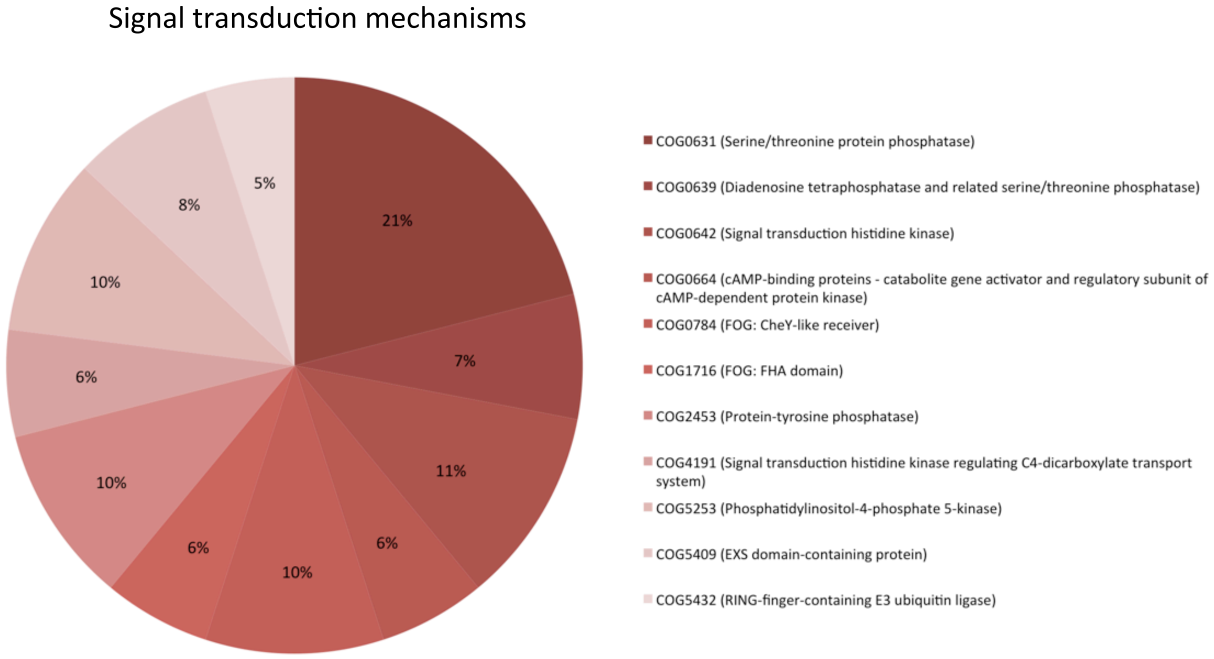

Figure S2. Percentage of second level COG terms of *Rafflesia cantleyi* transcriptome. (A) ‘Cell cycle control, cell division’. (B) ‘Signal transduction mechanisms’.
