# Supplementary material for: Transcriptome landscape of Rafflesia cantleyi floral buds reveals insights into the roles of transcription factors and phytohormones in flower development
Source: PLoS One. 2019 Dec 18;14(12):e0226338. doi: 10.1371/journal.pone.0226338 (PMC6919626; doi:10.1371/journal.pone.0226338)
Supplement: S3 Fig — (PDF) [file pone.0226338.s003.pdf]

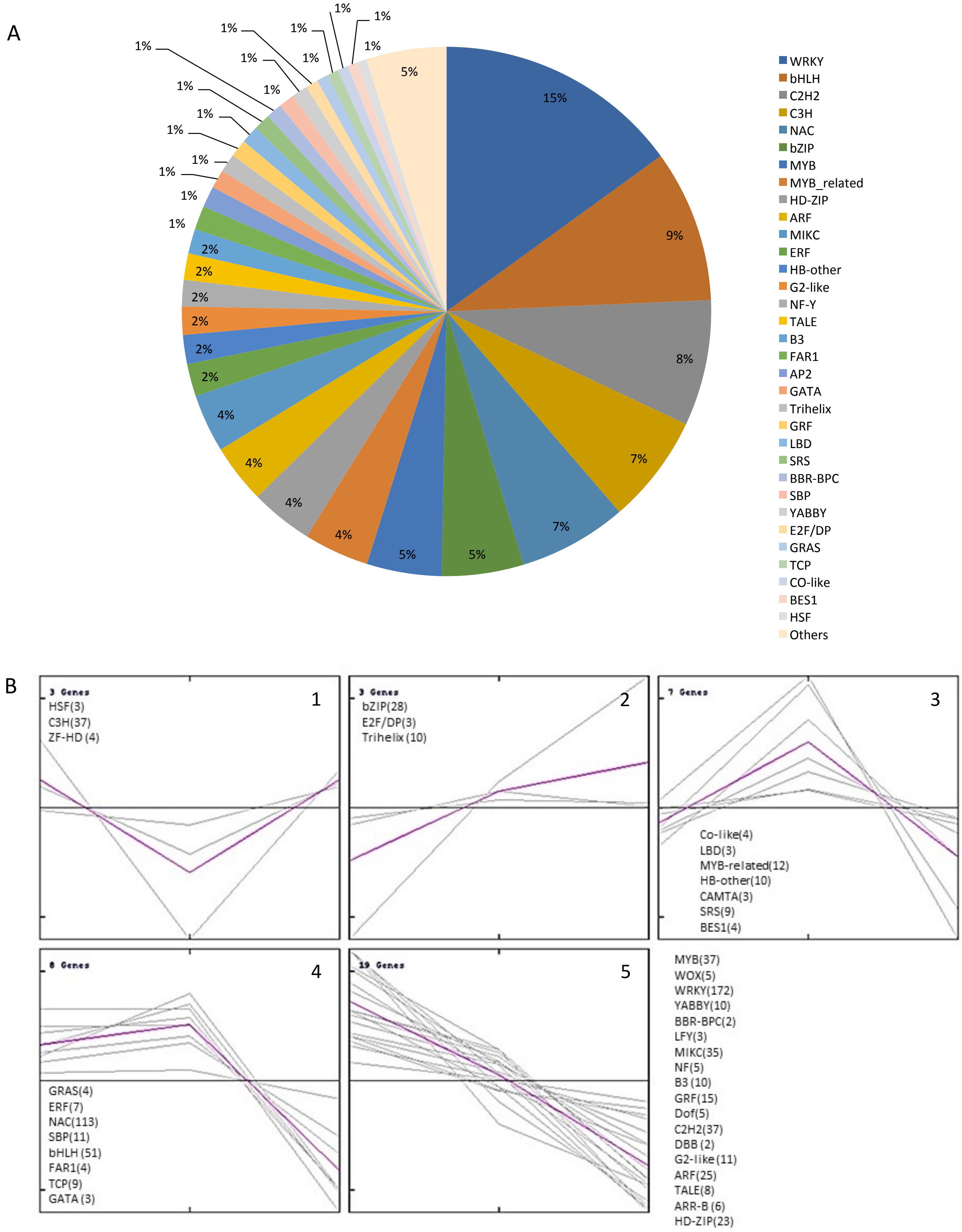

Figure S3. Abundance analysis and cluster analysis of transcription factors in *Rafflesia cantleyi* transcriptome. (A) Abundance analysis of transcription factors. (B) Cluster analysis of differentially expressed transcription factors from the expression profiles during the flowering process. Purple lines indicate the average expression level of transcripts grouped into the same cluster under different flowering stages.
