# Supplementary material for: Transcriptome landscape of Rafflesia cantleyi floral buds reveals insights into the roles of transcription factors and phytohormones in flower development
Source: PLoS One. 2019 Dec 18;14(12):e0226338. doi: 10.1371/journal.pone.0226338 (PMC6919626; doi:10.1371/journal.pone.0226338)
Supplement: S4 Fig — (PDF) [file pone.0226338.s004.pdf]

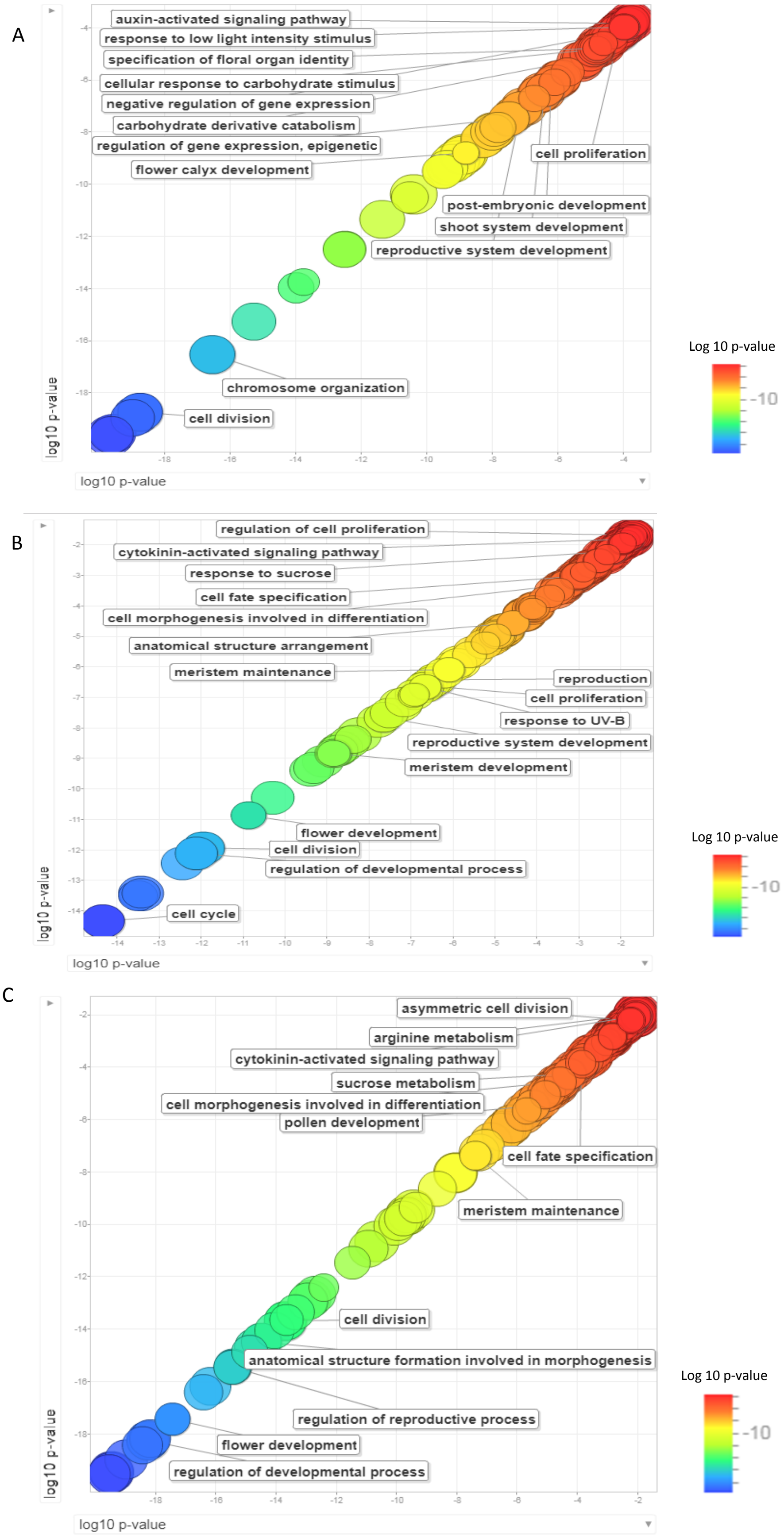

Figure S4. Scatterplot of enriched GO terms for DEGs using semantic clustering (REViGO). (A) FBS1 vs. FBS2, (B) FBS2 vs. FBS3, and (C) FBS1 vs. FBS3. Each circle represents a cluster of GO terms related to a similar process, and the size of the circle represents the number of GO terms grouped in that cluster. The color of the circles indicates the P-value of the GO enrichment analysis; red indicates the highest P-value and blue the lowest. The cutoff P-value for the GO enrichment analysis was set to 1E-03.
