# Supplementary material for: Transcriptome landscape of Rafflesia cantleyi floral buds reveals insights into the roles of transcription factors and phytohormones in flower development
Source: PLoS One. 2019 Dec 18;14(12):e0226338. doi: 10.1371/journal.pone.0226338 (PMC6919626; doi:10.1371/journal.pone.0226338)
Supplement: S1 Table — (PDF) [file pone.0226338.s005.pdf]

S1 Table. Primer sequences of reference genes and 12 selected genes for RT-qPCR analysis

Gene	Forward primer 5’ → 3’	Reverse primer 5’ → 3’
GAPDH	TGGCAAGAGGAGCAAGACAAT	GAAGGGTGGTGCCAAAAAGG
UBQ5	TTCTCCTCTACCCGTTCCTTG	TGTTGAGGCTTAGGGGAGGAA
FUL	TGCCTGCTTATCTTGTTCTCG	TCGCCCCCTCACCCATAAAT
AP2	ACGAGAGAGGGAGATGATGTG	GCCTGCCCTGTGCTGTATT
LFY	CGAGCACCCAGTACCCTTCT	GTTGGTTGGGGGTTGAGGA
AP3	TCTATCTTGCCCCGTCCCA	TTCCTGAAAGCTCCTCGTGT
SEP1	ACCTGCCAAAGAACTAGAGA	CAATTCCTTAGTGTTCAAAGGGC
SVP	TCTGTGATCGCTTGTTTCACC	CCTTCTCTCTCGCCATCACC
UFO	AACAAGGGGCAACAAGCATT	AATATGAGGGAGCAGTCGGT
FCA	GCTGTACTTGTTGCTGCTGTT	TGTAACTGGACAGAGCATACCTC
EMF2	TTCGACGTGGTTTTGCTCGT	GGGAAACTGGAGGAACAGCC
PIN1	GACTAGAGCGATGGGCAAGG	GGCGATGAGATTCCTTACGGG
GA2OX8	AACGAAGACCCACAGGACATC	CCTTCAATACGTCGCTGGAAA
CKX7	TGCTCGCACAAGTAGAGTCG	TACGCAATCATGTCGCAGTG
