# Supplementary material for: Transcriptome landscape of Rafflesia cantleyi floral buds reveals insights into the roles of transcription factors and phytohormones in flower development
Source: PLoS One. 2019 Dec 18;14(12):e0226338. doi: 10.1371/journal.pone.0226338 (PMC6919626; doi:10.1371/journal.pone.0226338)
Supplement: S2 Table — (PDF) [file pone.0226338.s006.pdf]

S2 Table. Functional annotation of *Rafflesia cantleyi* transcripts against Nr, Swiss-Prot, TAIR, PFAM, GO, COG and KEGG databases

**Database**	**Number of annotated transcripts**	**Percentage (%)**
Nr	31,444	35.0
Swiss-Prot	21,781	24.3
TAIR	25,970	28.9
PFAM-TMHMM	5,069	5.6
GO	28,922	32.3
COG	7,172	7.9
KEGG	5,850	6.5
