# Supplementary material for: Transcriptome landscape of Rafflesia cantleyi floral buds reveals insights into the roles of transcription factors and phytohormones in flower development
Source: PLoS One. 2019 Dec 18;14(12):e0226338. doi: 10.1371/journal.pone.0226338 (PMC6919626; doi:10.1371/journal.pone.0226338)
Supplement: S3 Table — (PDF) [file pone.0226338.s007.pdf]

S3 Table. GO classifications of transcripts

Ontology	Class	Number of transcripts
Biological process	Metabolic process	16950
Biological process	Cellular process	16346
Biological process	Single-organism process	11855
Biological process	Biological regulation	5762
Biological process	Response to stimulus	5515
Biological process	Cellular component organization or biogenesis	5221
Biological process	Regulation of biological process	5095
Biological process	Localization	3732
Biological process	Developmental process	3668
Biological process	Multicellular organismal process	3404
Biological process	Reproduction	2596
Biological process	Reproductive process	2432
Biological process	Multi-organism process	1443
Biological process	Signaling	1370
Biological process	Negative regulation of biological process	1349
Biological process	Positive regulation of biological process	1065
Biological process	Growth	853
Biological process	Immune system process	555
Biological process	Biological adhesion	140
Biological process	Rhythmic process	78
Cellular component	Cell	15385
Cellular component	Cell part	15345
Cellular component	Organelle	12145
Cellular component	Membrane	6977
Cellular component	Organelle part	4853
Cellular component	Macromolecular complex	4773
Cellular component	Membrane part	4321
Cellular component	Membrane-enclosed lumen	1447
Cellular component	Extracellular region	687
Cellular component	Cell junction	624
Cellular component	Symplast	623
Cellular component	Supramolecular complex	268
Cellular component	Virion	91
Cellular component	Virion part	88
Molecular function	Binding	14992
Molecular function	Catalytic activity	12510
Molecular function	Transporter activity	1540
Molecular function	Structural molecule activity	574
Molecular function	Nucleic acid binding transcription factor activity	564

Molecular function	Molecular function regulator	293
Molecular function	Signal transducer activity	239
Molecular function	Antioxidant activity	120
Molecular function	Electron carrier activity	111
Molecular function	Transcription factor activity, protein binding	102
Molecular function	Molecular transducer activity	101
Molecular function	Nutrient reservoir activity	24
