# Supplementary material for: Transcriptome landscape of Rafflesia cantleyi floral buds reveals insights into the roles of transcription factors and phytohormones in flower development
Source: PLoS One. 2019 Dec 18;14(12):e0226338. doi: 10.1371/journal.pone.0226338 (PMC6919626; doi:10.1371/journal.pone.0226338)
Supplement: S4 Table — (PDF) [file pone.0226338.s008.pdf]

S4 Table. KEGG classifications of transcripts

KEGG category	KEGG pathway	Pathway ID	Count
Metabolism	Purine metabolism	ko00230	3488
Metabolism	Thiamine metabolism	ko00730	1865
Metabolism	Biosynthesis of antibiotics	ko01130	960
Metabolism	Pyrimidine metabolism	ko00240	411
Metabolism	Aminobenzoate degradation	ko00627	372
Metabolism	Drug metabolism - other enzymes	ko00983	328
Metabolism	Starch and sucrose metabolism	ko00500	300
Metabolism	Inositol phosphate metabolism	ko00562	291
Metabolism	Other glycan degradation	ko00511	284
Metabolism	Oxidative phosphorylation	ko00190	230
Metabolism	Glycolysis / Gluconeogenesis	ko00010	219
Metabolism	Glycerophospholipid metabolism	ko00564	212
Metabolism	Alanine, aspartate and glutamate metabolism	ko00250	207
Metabolism	Amino sugar and nucleotide sugar metabolism	ko00520	200
Metabolism	Pentose and glucuronate interconversions	ko00040	195
Metabolism	Pyruvate metabolism	ko00620	192
Metabolism	Arginine biosynthesis	ko00220	191
Metabolism	Galactose metabolism	ko00052	178
Metabolism	Phenylpropanoid biosynthesis	ko00940	178
Metabolism	Cysteine and methionine metabolism	ko00270	173
Metabolism	Phenylalanine, tyrosine and tryptophan biosynthesis	ko00400	165
Metabolism	Glycerolipid metabolism	ko00561	157
Metabolism	Lysine degradation	ko00310	150
Metabolism	Fatty acid degradation	ko00071	147
Metabolism	Glycine, serine and threonine metabolism	ko00260	147
Metabolism	Arginine and proline metabolism	ko00330	143

Metabolism	Phenylalanine metabolism	ko00360	134
Metabolism	Fructose and mannose metabolism	ko00051	131
Metabolism	beta-Alanine metabolism	ko00410	122
Metabolism	Methane metabolism	ko00680	116
Metabolism	Fatty acid biosynthesis	ko00061	115
Metabolism	Valine, leucine and isoleucine degradation	ko00280	114
Metabolism	Propanoate metabolism	ko00640	112
Metabolism	Folate biosynthesis	ko00790	110
Metabolism	Alpha-linolenic acid metabolism	ko00592	109
Metabolism	Tyrosine metabolism	ko00350	108
Metabolism	Carbon fixation in photosynthetic organisms	ko00710	107
Metabolism	Nitrogen metabolism	ko00910	106
Metabolism	Carbon fixation pathways in prokaryotes	ko00720	103
Metabolism	Nicotinate and nicotinamide metabolism	ko00760	102
Metabolism	Tryptophan metabolism	ko00380	100
Metabolism	Ascorbate and aldarate metabolism	ko00053	96
Metabolism	Porphyrin and chlorophyll metabolism	ko00860	94
Metabolism	Glyoxylate and dicarboxylate metabolism	ko00630	86
Metabolism	Pentose phosphate pathway	ko00030	85
Metabolism	Sulfur metabolism	ko00920	83
Metabolism	Isoquinoline alkaloid biosynthesis	ko00950	80
Metabolism	Biosynthesis of unsaturated fatty acids	ko01040	78
Metabolism	Sphingolipid metabolism	ko00600	78
Metabolism	Drug metabolism - cytochrome P450	ko00982	77
Metabolism	Ether lipid metabolism	ko00565	75
Metabolism	Terpenoid backbone biosynthesis	ko00900	71
Metabolism	Butanoate metabolism	ko00650	70
Metabolism	Histidine metabolism	ko00340	68
Metabolism	Glutathione metabolism	ko00480	66

Metabolism	Tropane, piperidine and pyridine alkaloid biosynthesis	ko00960	66
Metabolism	Lysine biosynthesis	ko00300	64
Metabolism	Limonene and pinene degradation	ko00903	60
Metabolism	Pantothenate and CoA biosynthesis	ko00770	60
Metabolism	Metabolism of xenobiotics by cytochrome P450	ko00980	59
Metabolism	Glycosaminoglycan biosynthesis - HS/Hep	ko00534	58
Metabolism	Flavonoid biosynthesis	ko00941	54
Metabolism	N-Glycan biosynthesis	ko00510	54
Metabolism	One carbon pool by folate	ko00670	54
Metabolism	Retinol metabolism	ko00830	54
Metabolism	Fatty acid elongation	ko00062	53
Metabolism	Glycosaminoglycan biosynthesis - CS/DS	ko00532	52
Metabolism	D-Glutamine and D-glutamate metabolism	ko00471	50
Metabolism	Steroid hormone biosynthesis	ko00140	50
Metabolism	Cyanoamino acid metabolism	ko00460	49
Metabolism	Monobactam biosynthesis	ko00261	47
Metabolism	Various types of N-glycan biosynthesis	ko00513	45
Metabolism	Caprolactam degradation	ko00930	44
Metabolism	Riboflavin metabolism	ko00740	44
Metabolism	Citrate cycle (TCA cycle)	ko00020	43
Metabolism	Linoleic acid metabolism	ko00591	43
Metabolism	Benzoate degradation	ko00362	42
Metabolism	Chloroalkane and chloroalkene degradation	ko00625	40
Metabolism	Novobiocin biosynthesis	ko00401	40
Metabolism	Selenocompound metabolism	ko00450	39
Metabolism	Geraniol degradation	ko00281	38
Metabolism	Arachidonic acid metabolism	ko00590	36
Metabolism	Glycosaminoglycan degradation	ko00531	36
Metabolism	Glycosphingolipid biosynthesis - ganglio series	ko00604	36

Metabolism	Streptomycin biosynthesis	ko00521	34
Metabolism	Valine, leucine and isoleucine biosynthesis	ko00290	34
Metabolism	Biotin metabolism	ko00780	33
Metabolism	Ubiquinone and other terpenoid-quinone biosynthesis	ko00130	31
Metabolism	Insect hormone biosynthesis	ko00981	30
Metabolism	Carbapenem biosynthesis	ko00332	26
Metabolism	Steroid biosynthesis	ko00100	26
Metabolism	Phosphonate and phosphinate metabolism	ko00440	25
Metabolism	C5-branched dibasic acid metabolism	ko00660	23
Metabolism	Cutin, suberine and wax biosynthesis	ko00073	21
Metabolism	Glycosylphosphatidylinositol (GPI)-anchor biosynthesis	ko00563	21
Metabolism	Diterpenoid biosynthesis	ko00904	20
Metabolism	Neomycin, kanamycin and gentamicin biosynthesis	ko00524	20
Metabolism	Taurine and hypotaurine metabolism	ko00430	17
Metabolism	Caffeine metabolism	ko00232	16
Metabolism	Flavone and flavonol biosynthesis	ko00944	16
Metabolism	Glycosphingolipid biosynthesis - globo and isoglobo series	ko00603	16
Metabolism	Atrazine degradation	ko00791	14
Metabolism	Lipopolysaccharide biosynthesis	ko00540	12
Metabolism	Vitamin B6 metabolism	ko00750	12
Metabolism	Mannose type O-glycan biosynthesis	ko00515	11
Metabolism	Steroid degradation	ko00984	11
Metabolism	Zeatin biosynthesis	ko00908	11
Metabolism	Naphthalene degradation	ko00626	10
Metabolism	Ethylbenzene degradation	ko00642	7
Metabolism	Styrene degradation	ko00643	7
Metabolism	Aflatoxin biosynthesis	ko00254	6

Metabolism	Betalain biosynthesis	ko00965	5
Metabolism	Photosynthesis	ko00195	5
Metabolism	Stilbenoid, diarylheptanoid and gingerol biosynthesis	ko00945	5
Metabolism	Glucosinolate biosynthesis	ko00966	4
Metabolism	Indole alkaloid biosynthesis	ko00901	4
Metabolism	Synthesis and degradation of ketone bodies	ko00072	4
Metabolism	Anthocyanin biosynthesis	ko00942	3
Metabolism	Monoterpenoid biosynthesis	ko00902	3
Metabolism	Penicillin and cephalosporin biosynthesis	ko00311	3
Metabolism	Primary bile acid biosynthesis	ko00120	3
Metabolism	Sesquiterpenoid and triterpenoid biosynthesis	ko00909	3
Metabolism	Acarbose and validamycin biosynthesis	ko00525	2
Metabolism	Biosynthesis of ansamycins	ko01051	2
Metabolism	Biosynthesis of vancomycin group antibiotics	ko01055	2
Metabolism	Chlorocyclohexane and chlorobenzene degradation	ko00361	2
Metabolism	D-Arginine and D-ornithine metabolism	ko00472	2
Metabolism	Lipoic acid metabolism	ko00785	2
Metabolism	Other types of O-glycan biosynthesis	ko00514	2
Metabolism	Polyketide sugar unit biosynthesis	ko00523	2
Metabolism	Toluene degradation	ko00623	2
Metabolism	Biosynthesis of siderophore group nonribosomal peptides	ko01053	1
Metabolism	D-Alanine metabolism	ko00473	1
Metabolism	Fluorobenzoate degradation	ko00364	1
Metabolism	Glycosphingolipid biosynthesis - lacto and neolacto series	ko00601	1
Metabolism	Peptidoglycan biosynthesis	ko00550	1
Organismal Systems	T cell receptor signaling pathway	ko04660	239
Organismal Systems	Th1 and Th2 cell differentiation	ko04658	203

Environmental Information Processing	Phosphatidylinositol signaling system	ko04070	219
Environmental Information Processing	mTOR signaling pathway	ko04150	34
Genetic Information Processing	Aminoacyl-tRNA biosynthesis	ko00970	63
