# Supplementary material for: Transcriptome landscape of Rafflesia cantleyi floral buds reveals insights into the roles of transcription factors and phytohormones in flower development
Source: PLoS One. 2019 Dec 18;14(12):e0226338. doi: 10.1371/journal.pone.0226338 (PMC6919626; doi:10.1371/journal.pone.0226338)
Supplement: S5 Table — (PDF) [file pone.0226338.s009.pdf]

S5 Table. KEGG pathways enrichment analysis of DEGs

KEGG Pathway	Input number	Background number	P-value	Corrected P-value
**FBS1-FBS2**				
Homologous recombination	28	56	3.5E-09	1.6E-07
Arginine and proline metabolism	28	76	5.8E-07	1.5E-05
RNA degradation	32	104	2.4E-06	5.2E-05
Alpha-linolenic acid metabolism	15	31	2.1E-05	3.0E-04
Other glycan degradation	11	18	5.4E-05	6.9E-04
Nucleotide excision repair	22	69	5.7E-05	7.2E-04
Lysine biosynthesis	10	16	1.0E-04	1.2E-03
DNA replication	17	50	0.000207	0.002181
C5-Branched dibasic acid metabolism	7	10	7.2E-04	6.5E-03
**FBS2-FBS3**				
RNA degradation	52	104	5.44E-08	2.08E-06
Alanine, aspartate and glutamate metabolism	33	48	7E-08	3E-06
Arginine and proline metabolism	37	76	7E-06	1E-04
Alpha-linolenic acid metabolism	22	31	8E-06	1E-04
Lysine biosynthesis	12	16	6E-04	7E-03
Nicotinate and nicotinamide metabolism	11	14	8E-04	8E-03
Nitrogen metabolism	20	41	8E-04	8E-03
Propanoate metabolism	13	22	2E-03	2E-02
Nucleotide excision repair	26	69	0.003097	0.023818
Diterpenoid biosynthesis	11	18	3E-03	3E-02
Sulfur relay system	9	14	0.006324	0.042863
Beta-alanine metabolism	17	41	7E-03	5E-02
**FBS1-FBS3**				
Beta-alanine metabolism	34	41	1.05E-07	2.46E-06

RNA degradation	53	104	4.33E-06	7.30E-05
Alanine, aspartate and glutamate metabolism	32	48	6.96E-06	0.000109
Arginine and proline metabolism	41	76	1.87E-05	0.00026
Nicotinate and nicotinamide metabolism	14	14	0.000170853	0.001843096
Alpha-linolenic acid	20	31	0.000486	0.004557
